# CAR-adapted *PIK3CD* base editing enhances T cell anti-tumor potency

**DOI:** 10.1038/s43018-025-01099-7

**Published:** 2026-01-06

**Authors:** Philip Bucher, Nadine Brückner, Jule Kortendieck, Melanie Grimm, Jan T. Schleicher, Karlotta Bartels, Steffen Hardy, Martina Rausch, Hannah Wurzer, Meike Thiemann, Celina May, Mara Mitstorfer, Dennis Letzgus, Julia Quach, Carolin Schneider, Denis A. Ispan, Irene Gonzalez-Menendez, Nayan Jain, Yu-Jui Ho, Jiangqing Chen, Francisco J. Sánchez-Rivera, Jie Sun, Leticia Quintanilla-Martinez, Christoph Trautwein, Bettina Weigelin, Manfred Claassen, Michel Sadelain, Judith Feucht, Josef Leibold

**Affiliations:** 1 Cluster of Excellence iFIT (EXC2180) ‘Image-guided and Functionally Instructed Tumor Therapies’, University of Tübingen, Tübingen, Germany; 2 Department of Pediatric Hematology, Oncology, Gastroenterology, Nephrology and Rheumatology, University Children’s Hospital, University of Tübingen, Tübingen, Germany; 3 Department of Medical Oncology and Pneumology, University Hospital Tübingen, Tübingen, Germany; 4 Department of Internal Medicine I, University Hospital Tübingen, Tübingen, Germany; 5 M3 Research Center for Malignome, Metabolome and Microbiome, Faculty of Medicine, University of Tübingen, Tübingen, Germany; 6 Department of Computer Science, University of Tübingen, Tübingen, Germany; 7 Institute for Bioinformatics and Medical Informatics, University of Tübingen, Tübingen, Germany; 8 Werner Siemens Imaging Center, Department of Preclinical Imaging and Radiopharmacy, Eberhard Karls University, Tübingen, Germany; 9 Core Facility Metabolomics, Faculty of Medicine, University of Tübingen, Tübingen, Germany; 10 Institute of Pathology and Neuropathology, Comprehensive Cancer Center and University Hospital Tübingen, Tübingen, Germany; 11 Core Facility Histology, Faculty of Medicine, University of Tübingen, Tübingen, Germany; 12 Columbia Initiative in Cell Engineering and Therapy (CICET), Columbia University Irving Medical Center, Vagelos College of Physicians and Surgeons, New York, NY USA; 13 Cancer Biology and Genetics Program, Sloan Kettering Institute, Memorial Sloan Kettering Cancer Center, New York, NY USA; 14 Department of Cell Biology and Bone Marrow Transplantation Center of the First Affiliated Hospital, Zhejiang University School of Medicine, Hangzhou, China; 15 David H. Koch Institute for Integrative Cancer Research, Massachusetts Institute of Technology, Cambridge, MA USA; 16 Department of Biology, Massachusetts Institute of Technology, Cambridge, MA USA

**Keywords:** Cancer, Gene therapy, T cells, Functional genomics, Cell signalling

## Abstract

Insufficient functional T cell persistence impedes therapeutic success of chimeric antigen receptor (CAR) therapies. Here we performed a CAR-adapted base-editing screen of *PIK3CD*, a key regulator of T cell function, metabolism and fate. We identified point mutations that beneficially modulate CAR T cell profiles in 4-1BBz and 28z CAR T cells, respectively. We found that point mutations with differing effects on phosphatidylinositol-3-kinase delta (PI3Kδ) signaling activity were advantageous in distinct CAR contexts: The PI3Kδ-activating substitution E81K enhanced proliferation, metabolic fitness and effector function of 4-1BBz CARs, promoting long-term functional persistence and enhanced therapeutic efficacy in vivo. Conversely, the PI3Kδ-attenuating substitution L32P improved T cell memory formation and functionality of 28z CAR T cells. Together, our approach of rational optimization of activation-dependent signaling through targeted allelic reprogramming (ROADSTAR) illustrates the importance of CAR design-specific fine-tuning of intrinsic T cell signaling and demonstrates the potential of base editing for next-generation cellular therapies.

## Abstract (web summary)

By performing a CAR-adapted base-editing screen of phosphatidylinositol-3-kinase delta (PI3Kδ*, PIK3CD*), Bucher et al. identify mutations affecting endogenous PI3K–AKT signaling that enhances CAR T cell antitumor potency.

## Main

Chimeric antigen receptor (CAR) T cells are an effective treatment option against refractory or relapsed B cell malignancies and multiple myeloma^1–4^. US Food and Drug Administration-approved CAR designs incorporate CD28 or 4-1BB costimulatory molecules fused to the intracellular CD3z chain (‘28z’ or ‘BBz’) to reprogram T cell metabolism, function and phenotypic properties^2,5^. Despite high initial response rates in persons with hematological malignancies, CAR-mediated therapeutic efficacy is insufficient in a substantial number of persons and response rates against solid tumors are poor^6–10^.

Inherent T cell characteristics critically determine the therapeutic potency and clinical responses of CAR therapies^11–13^. In line with the pivotal role of signaling strength in T cell biology, the signaling domains incorporated in the CAR design thereby critically affect the characteristics and function of the cell product^14–18^. Furthermore, introduction of single-nucleotide mutations in the genome by CRISPR base editing can positively and negatively tune effector T cell responses^19–21^. To enable potent CAR T cell efficacy in vivo, we hypothesized that it may be crucial to modulate T cell activity in consideration of the unique signaling activities imparted by the CAR itself. Prototypic 28z-based CARs are associated with high effector function and a more rapid T cell expansion relative to BBz-based CARs but are more prone to T cell exhaustion and demonstrate reduced persistence in vivo^2,22^. These differences have been linked to the engagement of noncanonical NF-κB that promotes oxidative phosphorylation and improved memory formation in BBz CAR T cells and the PI3K–AKT pathway that fosters aerobic glycolysis and differentiation into an effector phenotype in the context of 28z CARs^5,23^.

Recent bioengineering strategies have leveraged T cell signaling modulation to advance CAR therapies. We and others have focused on the attenuation of CAR-mediated signaling strength to prevent early differentiation and exhaustion in 28z CAR T cells^17,24^. Conversely, approaches to augment signaling strength to boost T cell activation have been beneficial for BBz-based CAR T cells^15,25^. Other approaches involve the overexpression of complementary DNA (cDNA) or gene disruption to transcriptionally, epigenetically and/or metabolically rewire CAR T cell profiles^25–28^. However, these methods do not allow precisely calibrating the activation of downstream genes and pathways and uncoupling signaling from endogenous positive and negative feedback regulation.

We hypothesized that precision T cell engineering by rational optimization of activation-dependent signaling through targeted allelic reprogramming (ROADSTAR) would result in enhanced and optimized CAR T cell therapies.

Herein, we performed a CRISPR base-editing screen in the p110δ adaptor-binding domain (ABD) of *PIK3CD* (NCBI gene ID 5293) to identify point mutations resulting in functional improvement of prototypic 1928z and 19BBz CAR T cells. We identified specific mutations in this regulatory domain of p110δ that were enriched under repetitive antigen stimulation in either CAR design. Base-editing hits were associated with contrasting effects on PI3K–AKT signaling strength in 28z relative to BBz CAR T cells, thereby conferring favorable attributes to each CAR product while preserving their ascribed beneficial properties. The PI3Kδ-activating substitution E81K resulted in long-term functional persistence of BBz CAR T cells without evidence of malignant transformation and was associated with superior tumor control of hematological and solid tumors in vivo. Conversely, the substitution L32P attenuated PI3Kδ signaling and altered 28z CAR T cell properties toward favorable memory features, leading to enhanced survival in a metastatic neuroblastoma model in vivo. Together, these data nominate the induction of targeted point mutations in CAR T cells as an effective way to endogenously fine-tune signaling strength for enhanced long-term antitumor activity in vivo and underscore the importance of CAR-adapted precision engineering to unleash the full potential of CAR therapies.

## Base-editing screen to modulate PI3K activity in CAR T cells

**Fig. 1:**
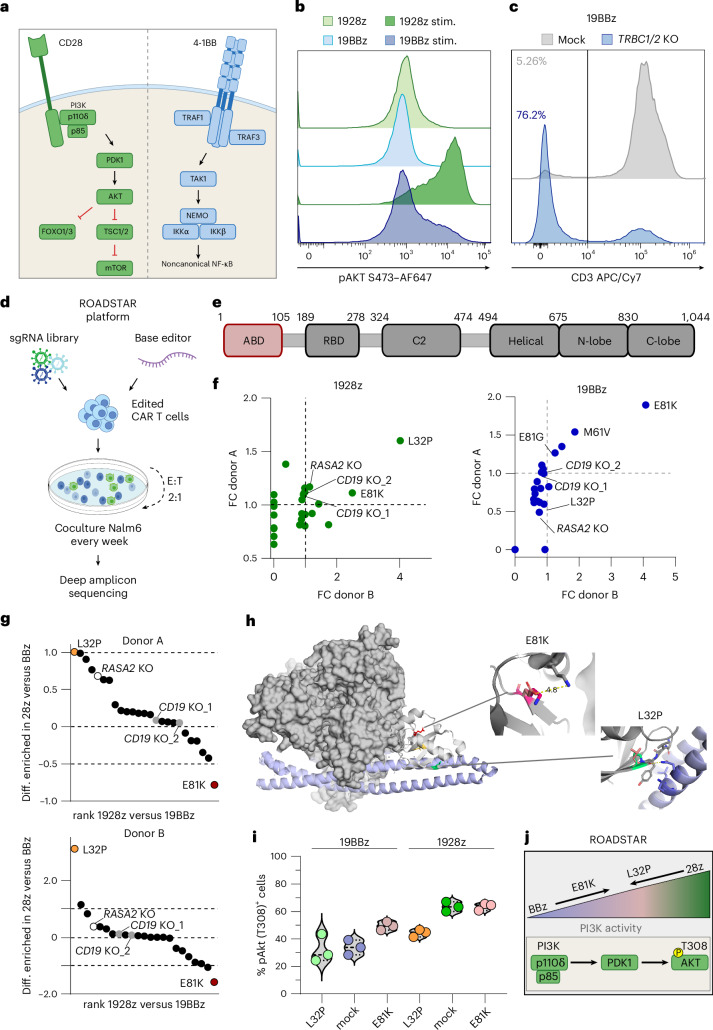
Base-editing screens of the ABD of PI3Kδ in 1928z and 19BBz CAR T cells identify beneficial point mutations. **a**, Schematic of CD28 and 4-1BB signaling in T cells. The drawing was adapted from a previous study^31^. **b**, Flow-cytometry-based analysis of pAKT S473 in 1928z and 19BBz CAR T cells without stimulation and 1 h after coculture with Nalm6 cells; stim., stimulated. Data are representative of *n* = 2 biological independent samples. **c**, Representative flow-cytometry-based analysis of CD3 surface expression in primary 19BBz CAR T cells after AncBE4max-mediated KO of *TRBC1* and *TRBC2* versus mock-electroporated CAR T cells. A modal *y*-axis representation is shown. Data are representative of *n* = 3 biologically independent samples. **d**, Schematic of the ABE and CBE screens performed in primary human 1928z and 19BBz CAR T cells. **e**, Schematic representation of the *PIK3CD* domains with indicated amino acid positions adapted from a previous study^31^.The screened ABD is highlighted in red. **f**, Fold-change enrichment of the respective point mutation in 1928z or 19BBz CAR T cells at the end of the screen relative to day 0 for biologically independent donor A and donor B. **g**, Differential enrichment of individual point mutations in 1928z versus 19BBz CAR T cells, calculated as the enrichment score in 1928z minus the corresponding score in 19BBz. Positive values indicate mutations enriched in 1928z over 19BBz CAR T cells, while negative values indicate enrichment in 19BBz over 1928z CAR T cells. Donors A and B are biologically independent samples. **h**, Structural model of the interaction of p110δ (gray) with p85 (light blue) (PDB 7JIS). The protein tertiary structure is shown for ABD and PIK3R1. The side chains for P32 (green) and K81 (red) substitutions are shown with their closest amino acid compared to the respective WT amino acid (light red). **i**, Flow-cytometry-based analysis of pAKT T308 in 19BBz and 1928z CAR T cells without (mock) and with the E81K and L32P substitutions 30 min after Nalm6 tumor cell stimulation at an E:T ratio of 1:1. Data are shown in technical triplicates from one representative donor of three biologically independent donors. **j**, Schematic representation of PI3K signaling activity in BBz-based and 28z-based CAR T cells, illustrating the impact of E81K and L32P substitutions on phosphorylation of AKT at T308. The drawing was adapted from a previous study^77^.

Costimulation provided by CD28 activates PI3K–AKT signaling and drives effector functions in T cells, whereas 4-1BB-mediated noncanonical NF-κB signaling promotes T cell survival (Fig. 1a)^29–31^. Cognizant of the key role of PI3K–AKT signaling in shaping (CAR) T cell fate and metabolism^32,33^, we sought to explore whether alteration of PI3K signaling strength could provide therapeutic benefit for CAR T cells. Phosphorylated (p)AKT (at residue S473) levels revealed marked differences in signaling strength upon antigen stimulation between 28z and BBz CAR T cells, with high pathway activity in 28z and moderate pAKT levels in BBz CAR T cells (Fig. 1b). In lymphocytes, PI3K signaling strength is mainly regulated by PI3Kδ, which is composed of the catalytic subunit p110δ (encoded by the gene *PIK3CD*) and the regulatory subunit p85 (encoded by the gene *PIK3R1*)^34^. We hypothesized that incorporation of single-point mutations in the ABD of *PIK3CD*—the domain that regulates the interaction of the catalytic subunit p110δ with the regulatory subunit p85—can be leveraged to calibrate PI3K–AKT signaling activity in CAR T cells.

We first established conditions to efficiently induce point mutations in primary human CAR T cells using the cytosine base editor (CBE; AncBE4max)^35^ and adenine base editor (ABE; ABEmax)^35^. The simultaneous delivery of CBE mRNA with a synthetic single guide RNA (sgRNA) targeting the T cell receptor β chain (*TRBC1* and *TRBC2*) by inducing a premature stop codon resulted in an effective disruption of the endogenous *TRBC* locus in CAR T cells. This led to a loss of cell-surface CD3 expression, enabling a fast and facile flow-cytometry-based readout that served as a reference for assessing editing efficiencies (Fig. 1c)^36^. This method yielded editing efficiencies of up to 75% and established a reliable basis for the implementation of our ROADSTAR platform (Fig. 1d), integrating a pooled base-editing screen to systematically evaluate the role of induced point mutations in the ABD of *PIK3CD* within CAR T cells.

**Extended Data Fig. 1:**
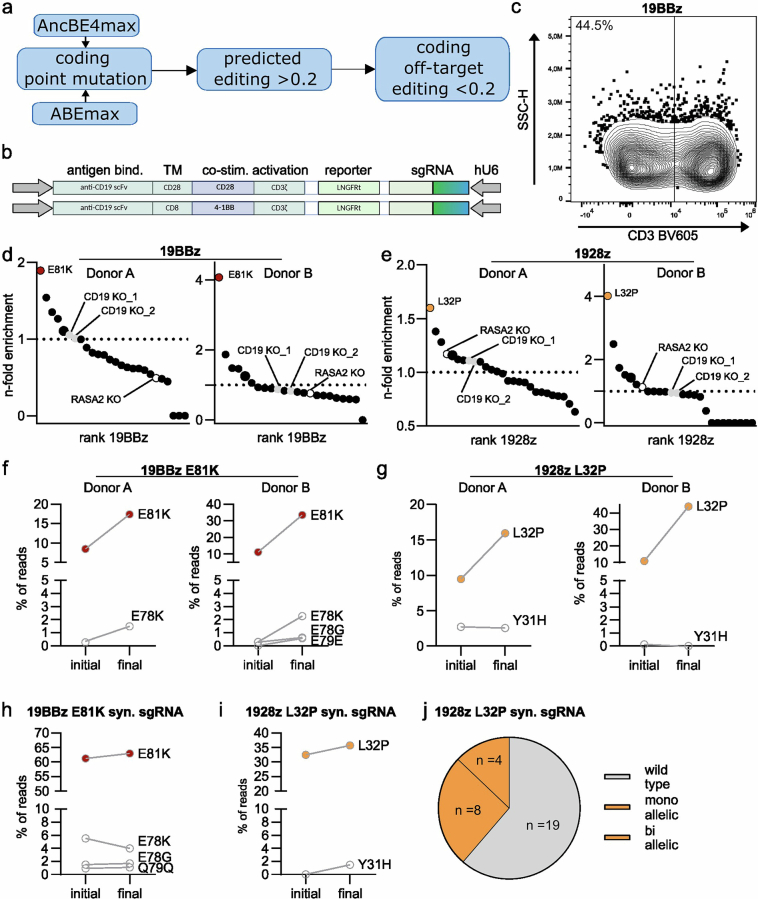
Base editing screens of the adaptor-binding domain (ABD) of PI3Kδ reveals differential enrichment of distinct point mutations in 1928z and 19BBz CAR T cells. **a**. Design of sgRNA library by BE-DICT online tool^78^. **b**. Schematic of the 19BBz or 1928z CAR construct including the sgRNA cassette. **c**. Representative flow cytometry-based analysis of CD3 surface expression in 19BBz CAR T cells after cytidine base editor (CBE; AncBE4max)-mediated knockout of TRBC1 and TRBC2; sgRNA is expressed on the CAR vector under a human U6 promoter as demonstrated in (**b**.). Data are representative for n = 3 biologically independent donors. **d**., **e**. n-fold enrichment of the respective point mutation in 19BBz (**d**.) and 1928z (**e**.) CAR T cells at the end of the screen relative to day 0 for biologically independent Donor A and Donor B. **f**., **g**. Total mutation frequency at day 0 (“initial”) and at the end point (“final”) for the E81K mutation in 19BBz CAR T cells (**f**.) and the L32P mutation in 1928z CAR T cells (**g**.) as identified in the screen by NGS analysis. Frequencies of co-occurring mutations near or within the editing window are shown as the proportion in all reads containing E81K (**f**.) and L32P (**g**.) editing, respectively. **h**.-**i**. Total mutation frequency in CAR T cells at day 0 (“initial”) and after three stimulations with Nalm6 tumor cells at an effector to target ratio of 2:1 (“final”). The E81K mutation in 19BBz CAR T cells (**h**.) and the L32P mutation in 1928z CAR T cells (**i**.), were introduced via synthetic sgRNAs. Co-occurring mutations are shown as a frequency of all reads covering the respective position. Data represents one donor. **j**. Number of analyzed single 1928z CAR T cell clones carrying either a monoallelic or biallelic L32P mutation, or no mutation (“wild type”), as determined by Sanger sequencing and quantified using EditR^61^. Data originate from n = 2 biologically independent donors.

We designed an sgRNA library of *n* = 34 sgRNAs predicted to induce a single-amino-acid change in the ABD of *PIK3CD* (https://bedict.forone.red/#/per-base) (Extended Data Fig. 1a), and included different control sgRNAs to guide cutoff values for determining hits and to calibrate results of the screen. An *RASA2*-targeting sgRNA served as a positive control given its role as a signaling checkpoint whose ablation enhances 1928z CAR T cell activity^37,38^. In addition, two sgRNAs predicted to result in a functional loss of CD19, which is absent in T cells, served as ‘neutral’ controls. To restrict gene editing to CAR-positive cells in the screening setup, we generated a combined CD19-specific CAR sgRNA γ-retroviral vector (Extended Data Fig. 1b) and confirmed efficient editing in this system after transfection of the cells with the respective base editor mRNA by assessing *TRBC* knockout (KO) efficiencies in primary CAR T cells (Extended Data Fig. 1c). Next, we transduced T cells of two individual healthy donors with the sgRNA library containing 1928z or 19BBz CAR constructs followed by electroporation with the respective base editor mRNA (Fig. 1d). Base-edited 1928z and 19BBz CAR T cell pools were evaluated in an in vitro CAR T cell ‘stress test’ with repetitive exposure to CD19-expressing Nalm6 leukemia cells (Fig. 1d). Abundance of each point mutation was determined by next-generation sequencing (NGS) before exposure of CAR T cells to the target cells and after repeated tumor stimulations, allowing us to determine individual edits that provide a functional advantage to CAR T cells through proliferative competition under antigen stimulation (Fig. 1d). As expected, the abundance of point mutations resulting in *CD19* KO did not significantly change during the 3-week coculture assay and the point mutation conferring a loss of *RASA2* was enriched in the context of 1928z CAR T cells. Importantly, results were consistent across both donors, confirming the robustness of our screening approach (Fig. 1e–g and Extended Data Fig. 1d,e).

In 19BBz CAR T cells, an E81K mutant showed the highest enrichment over the 3-week assay (Fig. 1f,g and Extended Data Fig. 1d,f). In 1928z CAR T cells, an L32P mutant was most strongly enriched after repetitive tumor stimulations (Fig. 1f,g and Extended Data Fig. 1e,g). Interestingly, the most highly enriched substitution within each CAR setting showed the strongest differential enrichment when comparing 1928z and 19BBz CARs (Fig. 1g), underscoring the difference in underlying signaling patterns afforded by the relevant costimulatory domains.

To predict potential functional consequences of the identified substitutions, we used the published crystal structure of PI3Kδ and overlaid the wild-type (WT) amino acid sequence with the respective virtually mutated sequences (Fig. 1h). The biochemical properties suggest that the insertion of the structural rigid proline instead of leucine might result in conformational changes that potentially affect the interaction site of the catalytic and the regulatory subunit of PI3Kδ located next to the substitution position 32 (Fig. 1h). The E81K substitution results in a charge reversal as glutamate is replaced by lysine. The proximity to other positively charged residues in the linker region next to E81 potentially results in notable structural changes, suggesting relevant effects on the interaction of p110δ with p85 and, subsequently, on the levels of PI3Kδ activity (Fig. 1h).

To evaluate the effect of the most enriched base edits on CAR T cell biology, we first confirmed successful editing induced by coelectroporation of T cells with base editor mRNA and the respective synthetic sgRNA followed by retroviral CAR transduction. This approach yielded high editing efficiencies, typically exceeding 50% for the E81K substitution in 19BBz CAR T cells (Extended Data Fig. 1h). Editing efficiencies for the L32P substitution in 1928z CAR T cells were typically around 30% and single-cell analysis revealed a mixture of clones harboring either monoallelic or biallelic edits (Extended Data Fig. 1i,j). Importantly, haplotype analysis did not reveal consistent enrichment of additional mutations co-occurring with the respective top hits E81K or L32P across donors, supporting on-target specificity and functional relevance of the identified top hits (Extended Data Fig. 1f–i).

To evaluate the effects of the L32P and E81K substitutions on PI3K–AKT signaling activity, we assessed the levels of pAKT (at residue T308) in edited CAR T cells upon antigen encounter. Induction of the E81K substitution resulted in a notable increase in pAKT in 19BBz CAR T cells but not to the extent of 1928z CAR T cells (Fig. 1i,j). Conversely, the L32P point mutant dampened the signaling response in 1928z CAR T cells as indicated by reduced pAKT levels (Fig. 1i,j).

## E81K substitution increases PI3K signaling activity and ameliorates BBz CAR T cells

**Extended Data Fig. 2:**
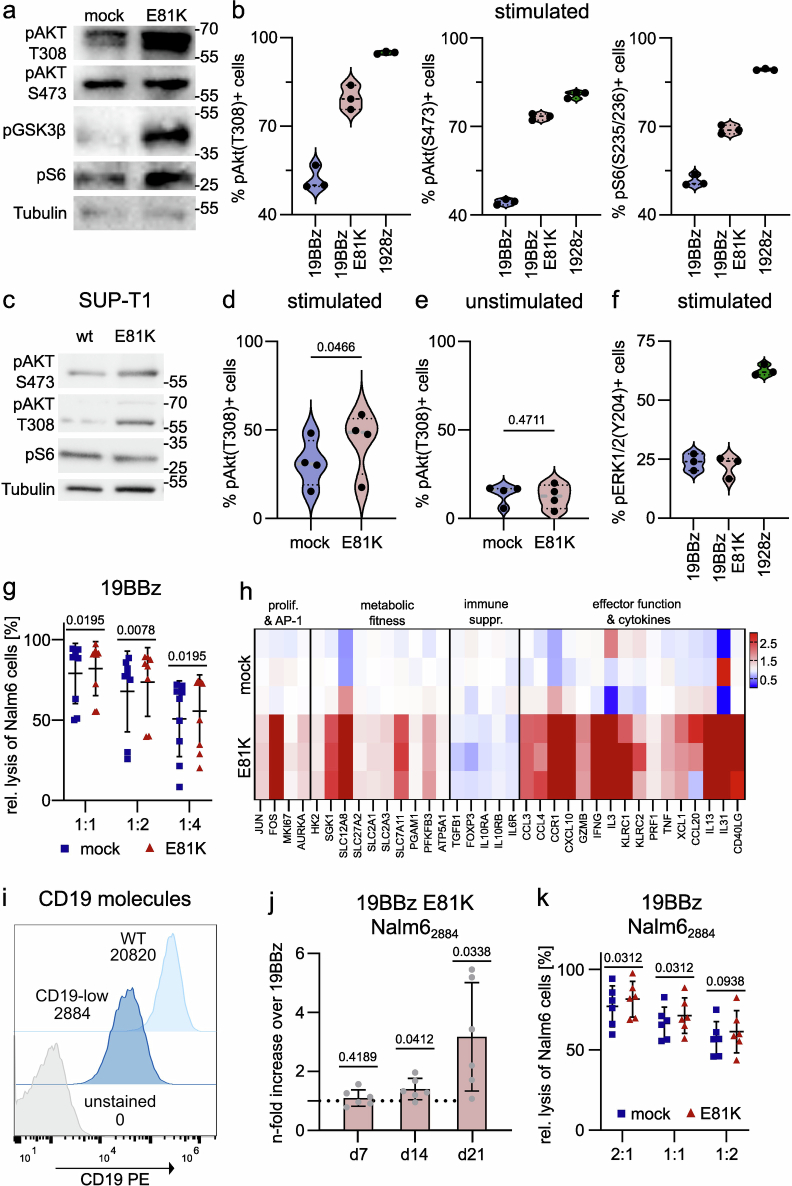
E81K editing modulates PI3K signaling and enhances cytotoxic capacity and antigen sensitivity in 19BBz CAR T cells. **a**. Immunoblot of primary 19BBz CAR T cells with TRBC1/2 KO (“mock”) or additional E81K editing (“E81K”) after 30 min of stimulation with Nalm6 at an effector to target (E:T) ratio of 1:1. p-AKT T308 and S473, p-GSK3β Ser9 and p-S6 S235/236 are shown, Tubulin served as loading control. Data represent one biological donor. **b**. Flow cytometry-based analysis of phosphorylated (p)-AKT T308, S473 and p-S6 S235/236 in 19BBz, E81K-edited 19BBz and 1928z CAR T cells, all with additional TRBC knockout (KO) after 1 h of stimulation with Nalm6 at an effector to target (E:T) ratio of 1:1. Data are shown as technical triplicates from one representative donor (out of three biologically independent donors). **c**. Immunoblot of single cell clones derived from unstimulated SUP-T1 cells either with the wild-type sequence (wt) or a homozygous E81K mutation. p-AKT T308 and S473, and p-S6 S235/236 are shown, Tubulin served as loading control. Data represent one independent experiment. **d**. Flow cytometry-based analysis of phosphorylated (p)-AKT T308 in control or E81K-edited TRBC KO 19BBz CAR T cells generated from T cells of pre-treated tumor patients 1 h after co-culture with Nalm6 cells (E:T 1:1; n = 4 biologically independent tumor patients, mean ± SD; two-sided paired Student’s *t*-test). **e**. Flow cytometry-based analysis of phosphorylated (p)-AKT T308 in unstimulated control or E81K-edited TRBC KO 19BBz CAR T cells generated from pre-treated tumor patients (n = 4 biologically independent tumor patients, mean ± SD; two-sided paired Student’s *t*-test). **f**. Flow cytometry-based analysis of phosphorylated (p)-ERK1/2 Y204 in TRBC KO 19BBz, E81K-edited TRBC KO 19BBz and TRBC KO 1928z CAR T cells 1 h after stimulation with Nalm6 cells. Data are shown in triplicates from one representative donor out of n = 3 biologically independent donors. **g**. Cytotoxic activity of TRBC KO 19BBz compared to TRBC KO E81K-edited 19BBz CAR T cells using an 18 h bioluminescence assay with luciferase-expressing Nalm6 wild type cells as targets for indicated effector to target (E:T) ratios (n = 9 biologically independent donors, mean ± SD; two-sided Wilcoxon matched-pairs signed rank test). **h**. Heatmap of genes of interest related to proliferation (prolif.), metabolic fitness, immune suppression (suppr.) and effector function comparing unstimulated E81K-edited (“E81K”) and control 19BBz CAR T cells (both with TRBC KO) (n = 3 technical replicates per group from one healthy donor) analyzed by bulk RNA sequencing; rows normalized to mean of control group. **i**. Flow cytometric analysis of CD19 expression levels in CD19-low und CD19 wild type Nalm6 cells expressing luciferase. CD19 surface counts were analyzed by Quantum™ Simply Cellular® beads. **j**. Antigen-dependent cell growth of E81K-edited 19BBz relative to control 19BBz CAR T cells after stimulation with CD19-low Nalm6_2884_ cells at an effector to target (E:T) ratio of 2:1 (n = 6 biologically independent donors, mean ± SD; two-sided one-sample t-test). **k**. Cytotoxic activity of TRBC KO 19BBz compared to TRBC KO E81K-edited 19BBz CAR T cells using an 18 h bioluminescence assay with luciferase-expressing CD19-low Nalm6 cells as targets for indicated effector to target (E:T) ratios (n = 6 biologically independent donors, mean ± SD; two-sided Wilcoxon matched-pairs signed rank test). Source data

Given the intermediate activation of the PI3K pathway in E81K-edited 19BBz CARs as compared to control 19BBz and 1928z CAR T cells, we examined effects on downstream signaling. Primary E81K-modified 19BBz CAR T cells showed increased phosphorylation of PI3K–AKT effectors relative to controls without reaching the levels achieved in 1928z CAR T cells (Extended Data Fig. 2a,b). These results were independently validated in CAR T cells generated from a SUP-T1 single-cell clone engineered to carry a homozygous E81K substitution and from patient-derived T cells (Extended Data Fig. 2c,d). Notably, increased pAKT induced by E81K was only detected upon antigen exposure of CAR T cells while basal pAKT activity remained similar to controls (Extended Data Fig. 2e), indicating that the E81K edit enhances signaling capacities only upon antigen stimulation. Other key signaling pathways in T cell activation such as the MAPK–ERK pathway remained unaffected by the E81K edit (Extended Data Fig. 2f).

**Fig. 2:**
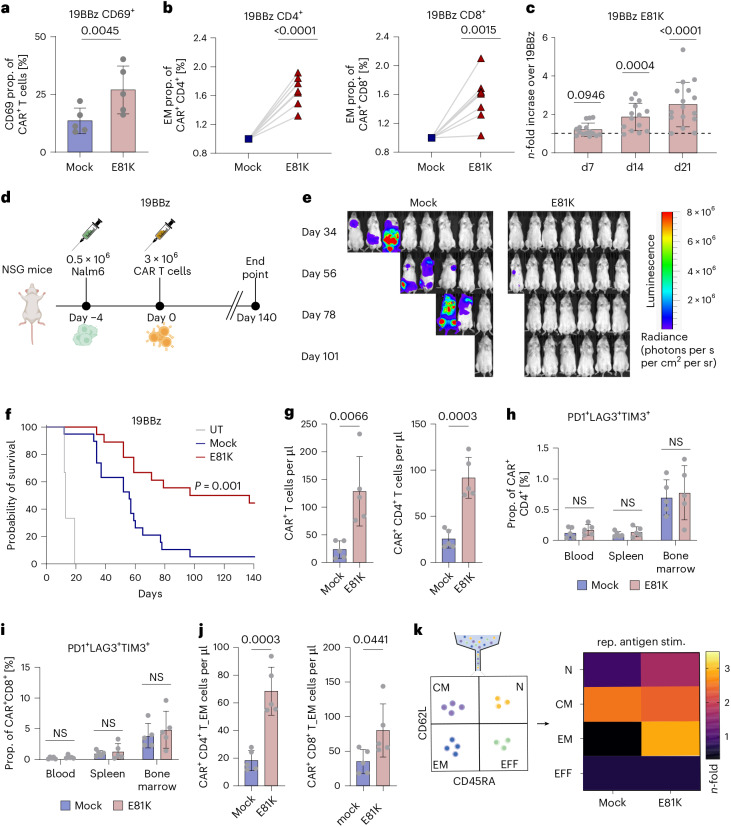
E81K enhances antitumor efficacy of 19BBz CAR T cells in vivo by increasing activation and effector memory profiles while preventing exhaustion. **a**, CD69 expression levels in 19BBz as compared to E81K-modified 19BBz (both with an additional *TRBC* KO) determined by flow cytometry without further antigen stimulation (*n* = 5 biologically independent donors, mean ± s.d.; two-sided paired Student’s *t*-test). **b**, Fold enrichment of the effector memory population (EM; CD62L^−^CD45RA^−^) in E81K-edited 19BBz relative to mock 19BBz CAR T cells (both *TRBC* KO) within the CD4^+^ (left) and CD8^+^ (right) CAR^+^ compartment. Statistical analysis was performed using a two-sided one-sample *t*-test (*n* = 8 biologically independent donors). **c**, *n*-fold CAR T cell expansion for E81K-edited relative to unmodified 19BBz (*TRBC* KO) CAR T cells 7 days after one (day 7), two (day 14) and three (day 21) stimulation(s) with Nalm6 cells (two-sided one-sample Wilcoxon test; *n* = 16 biologically independent donors, mean ± s.d.). **d**–**j**, Luciferase-expressing Nalm6 cells were i.v. injected into NSG mice followed by i.v. administration of untransduced T cells (UT) or 19BBz *TRBC*-KO CAR T cells without (mock) or with E81K substitution. **d**, Experimental timeline. **e**, Bioluminescence images of Nalm6-bearing mice (representative of **f**) treated with control 19BBz or E81K-edited 19BBz *TRBC*-KO CAR T cells (ventral view). **f**, Survival of the cohort (E81K 19BBz: *n* = 18, control 19BBz: *n* = 19 mice per group, UT: *n* = 3 mice per group; T cells from *n* = 4 unrelated, biologically independent healthy donors). A two-sided Mantel–Cox test was performed for statistical analysis. **g**, Absolute counts of 19BBz CAR T cells with and without the E81K substitution in blood and of CD4^+^ E81K-edited and control (mock) 19BBz CAR T cells in blood 16 days after CAR T cell isolation (*n* = 5 mice, mean ± s.d.; unpaired two-sided Student’s *t*-test). **h**,**i**. Flow cytometric analysis of TIM3^+^PD1^+^LAG3^+^ *TRBC*-KO 19BBz CAR T cells with and without E81K substitution for CD4^+^ (**h**) and CD8^+^ (**i**) T cells in blood, spleen and bone marrow (left to right) of mice 16 days after CAR T cell infusion (*n* = 5 mice, mean ± s.d.; two-sided unpaired Student’s *t*-test). **j**, Absolute CD4^+^ and CD8^+^ effector memory counts of control (mock) and E81K-edited 19BBz CAR T cells in peripheral blood 16 days after CAR T cell infusion (*n* = 5 mice, mean ± s.d.; unpaired two-sided Student’s *t*-test). **k**, Experimental schematic of 19BBz CAR T cell subsets (with or without additional E81K substitution) sorted on the basis of CD62L and CD45RA expression (naive, N; central memory, CM; effector memory, EM; effector, EFF). CAR T cells were subsequently exposed to repetitive tumor stimulations. Heat map demonstrating relative expansion of T cell subsets comparing unmodified and E81K-edited 19BBz CAR T cells after three rounds of antigen stimulation with Nalm6 cells. Data are representative of *n* = 2 biologically independent donors.

Consistent with these findings, the E81K substitution resulted in an increased fraction of CD69^+^ BBz CAR T cells (Fig. 2a) and in enhanced differentiation to an effector memory T cell state (Fig. 2b). These changes were associated with a considerably increased antigen-dependent proliferation capacity and a significantly enhanced cytolytic activity of E81K-edited relative to control 19BBz CAR T cells (Fig. 2c and Extended Data Fig. 2g). Transcriptional profiles confirmed upregulation of key genes related to cytokine production, effector function, proliferation and cell cycle in E81K-edited compared to mock BBz CAR T cells and indicated enhanced metabolic fitness induced by the E81K substitution (Extended Data Fig. 2h). Furthermore, the greater signaling response of E81K-edited BBz CAR T cells was associated with improved sensitivity to low antigen levels, resulting in superior proliferation and killing in antigen-low tumor settings (Extended Data Fig. 2i–k), a known limitation of prototypic 19BBz CARs^25,39^.

**Extended Data Fig. 3:**
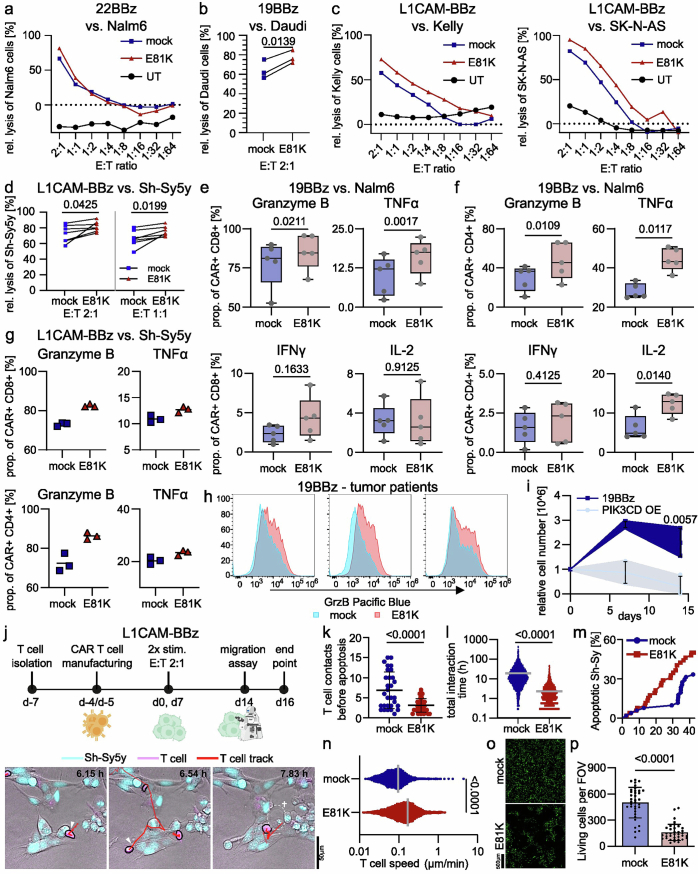
Increased effector function of E81K base-edited CAR T cells across different tumor entities and targets. **a**. Cytotoxic activity of mock CD22-specific BBz CAR T cells compared to E81K-edited 22BBz CAR T cells at first stimulations with Nalm6 cells using an 18 h bioluminescence assay with luciferase-expressing Nalm6 cells as targets. E:T, effector to target ratio (mean of n = 3 technical replicates, reproduced in two biologically independent donors). UT, untransduced. **b**. Cytotoxic activity of control mock 19BBz compared to E81K-edited 19BBz CAR T cells at first stimulation with Daudi cells using an 18 h bioluminescence assay with luciferase-expressing Daudi cells as targets at an E:T ratio of 2:1. E:T, effector to target ratio (n = 3 biologically independent donors; two-sided paired Student’s *t*-test). **c**. Cytotoxic activity of L1CAM-specific BBz CAR T cells with TRBC KO compared to E81K-edited L1CAM-BBz CAR T cells with TRBC KO at first tumor stimulation using an 18 h bioluminescence assay with luciferase-expressing Kelly and SK-N-AS cells as targets. E:T, effector to target ratio (mean of n = 3, reproduced in two biologically independent donors); UT, untransduced. **d**. Cytotoxic activity of mock L1CAM-BBz compared to E81K-edited L1CAM-BBz CAR T cells at first stimulation with luciferase-expressing Sh-Sy5y cells as targets, using an 18 h bioluminescence assay. E:T, effector to target ratio (n = 6 biologically independent donors, mean; two-sided paired Student’s *t*-test). **e**., **f**. Effector cytokine and Granzyme B production in CD8+ (**e**.) and CD4+ (**f**.) E81K-edited and mock 19BBz CAR T cells after stimulation with Nalm6 cells as measured by flow cytometry (n = 5 biologically independent donors; two-sided paired Student’s *t*-test). Center line shows the median, box represents 25th and 75th percentiles and whiskers minimum to maximum values. **g**. Granzyme B and TNFα production in CD8+ and CD4 + E81K-edited and mock L1CAM-BBz CAR T cells after a 4 h stimulation with Sh-Sy5y cells as measured by flow cytometry (n = 3 technical replicates). **h**. Flow cytometric analysis of Granzyme B (GrzB) levels in mock 19BBz and E81K-modified 19BBz CAR T cells generated from pre-treated biologically independent tumor patients (n = 3). Each plot represents one donor, modal representation. **i**. Antigen-dependent growth of 19BBz CARs with and without exogenous overexpression of *PIK3CD* during two repetitive stimulations with Nalm6 (n = 3 biologically independent donors, mean ± SD). **j**.-**p**. 3D collagen gel migration assay using H2B-GFP-expressing Sh-Sy5y as target cells and E81K-edited (“E81K”) or control L1CAM-BBz CAR T cells (both with TRBC knockout (KO)) as effector cells. Data represent one healthy donor. **j**. Upper panel showing schematic of the experimental timeline, lower panel showing representative images of T cell – tumor cell interactions from timelapse recordings in the 3D collagen cytotoxicity assay. T cell (magenta); GFP+ Sh-Sy5y neuroblastoma cells (blue); T cell migration tracks (red); tumor cell apoptosis following a T cell contact (white circle). **k**. Number of T cell contacts before apoptosis induction in Sh-Sy5y cells upon co-culture with mock and E81K-edited L1CAM-BBz CAR T cells (mean ± SD, two-sided unpaired t-test). Total interaction time of mock or E81K-modified L1CAM-BBz CAR T cells with Sh-Sy5y target cells (**l**.) (two-sided unpaired t-test), the percentage of apoptotic events for GFP+ Sh-Sy5y cells over time (**m**.) and T cell speed (**n**.) in mock and E81K-edited L1CAM-BBz CAR conditions (two-sided unpaired t-test). Exemplary field of view (**o**.) and tumor cell count per field of view (*n* = 36) (**p**.) of Sh-Sy5y H2B-GFP+ cells after 96 h of co-culture with CAR T cells and treatment as described in (**j**.) (mean ± SD, two-sided unpaired t-test).

To expand the applicability of E81K-edited BBz CAR T cells, we assessed their antitumor activity across multiple disease models. The E81K substitution enhanced BBz CAR functionality against a broad range of targets and malignancies, including hematological and solid tumors. This benefit was observed in CAR T cells generated from both healthy donors and heavily pretreated patients with cancer, underscoring the translational potential of E81K-edited BBz CAR T cells (Extended Data Fig. 3a–h).

The improved functionality of BBz-based CAR T cells relied on the endogenous modulation of PI3K signaling as exogenous cDNA-mediated overexpression of p110δ impaired antitumor activity of 19BBz CAR T cells (Extended Data Fig. 3i), highlighting the importance of precisely balancing PI3K signaling in CAR T cells.

To further delineate CAR T cell effector function at the single-cell level, we performed live-cell imaging of E81K-edited or control L1CAM-BBz CAR T cells in three-dimensional (3D) collagen gel cocultures with endogenously L1CAM-expressing Sh-Sy5y neuroblastoma cells (Extended Data Fig. 3j). E81K-edited CAR T cells required fewer and shorter interactions with target cells to induce apoptosis (Extended Data Fig. 3k,l), resulting in substantially higher and faster induction of target cell apoptosis (Extended Data Fig. 3m). Additionally, long-term time-lapse microscopy revealed an improved migratory phenotype of base-edited CAR T cells demonstrating increased speed (Extended Data Fig. 3n). Altogether, these studies indicate that E81K-edited CAR T cells are more efficient in tumor cell killing, reinforcing their overall increased functionality and resulting in improved tumor control relative to nonedited CAR T cells (Extended Data Fig. 3o,p).

## E81K-edited BBz CAR T cells provide long-term antitumor efficacy in vivo without increased safety risk

**Extended Data Fig. 4:**
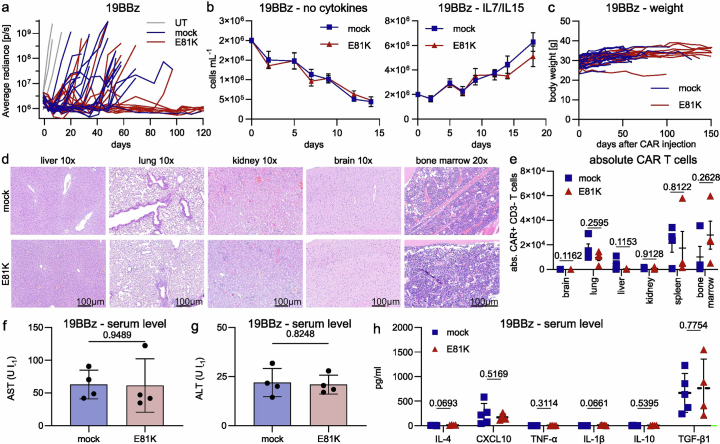
E81K-modified 19BBz CAR T cells are safe and do not promote malignant transformation in vivo. **a**. Tumor growth in the Nalm6 experiment described in Fig. 2d was monitored by bioluminescence imaging (E81K 19BBz: n = 12, control 19BBz: n = 13 mice per group, UT: n = 3 mice per group; T cells from n = 3 biologically independent healthy donors). **b**. Relative cell growth of E81K-modified and mock 19BBz CAR T cells without (left panel) and with (right panel) IL-7/IL-15 support (technical triplicates; mean ± SD). **c**. Body weight of NSG mice that were i.v. injected with Nalm6 cells followed by treatment with 3e6 19BBz CAR T cells (TRBC KO) with and without E81K editing during an observation period of 150 days. Data represent n = 19 mice per group treated with CAR T cells from n = 4 biologically independent healthy donors. **d**. Representative H&E stainings from livers, lungs, kidneys, brains, and bone marrows 66 days after i.v. infusion of E81K-modified or unmodified 19BBz CAR T cells (TRBC KO) in Nalm6-bearing NSG mice (n = 4 mice per group; scale bar size as indicated). **e**. Flow cytometric analysis of absolute CAR T cell numbers in brain, lung, liver, kidney, spleen and bone marrow 66 days after administration of E81K-modified or unmodified 19BBz CAR T cells (TRBC KO) in Nalm6-bearing mice (n = 4 mice per group; mean + SD; two-sided unpaired Student’s *t*-test). **f**., **g**. Serum levels of AST (**f**.) and ALT (**g**.) 66 days after infusion of 19BBz CAR T cells with and without E81K modification in Nalm6-bearing mice (n = 4 mice; mean ± SD; two-sided unpaired Student’s *t*-test). **h**. Cytokine serum levels 16 days after CAR T cell infusion in Nalm6-bearing mice as measured by multiplexed ELISA (n = 5 mice, mean ± SD; two-sided unpaired Student’s *t*-test).

To test whether these enhanced phenotypic and functional properties of E81K-edited CAR T cells translate into superior tumor control in vivo, we turned to the well-established preclinical Nalm6 leukemia mouse model (Fig. 2d). To prevent graft-versus-host disease and enable long-term monitoring of mice, we additionally applied base editing to induce *TRBC* KO in E81K-edited and mock CAR T cells. E81K-edited 19BBz CAR T cells elicited enhanced tumor control compared to control CAR T cells and could induce long-term remissions in mice, resulting in significantly improved survival (Fig. 2e,f and Extended Data Fig. 4a).

Given recent concerns of toxicity or malignant transformation^40^, we performed in-depth safety analyses of E81K-edited CAR T cells. Long-term culture of E81K-modified 19BBz CARs without or with cytokine support did not show aberrant cell growth in the absence of antigen stimulation (Extended Data Fig. 4b). Likewise, mice treated with E81K-edited BBz CAR T cells exhibited no signs of morbidity or changes in weight in long-term follow up studies (Extended Data Fig. 4c). Furthermore, necropsies of treated mice 66 days after CAR T cell infusion did not identify aberrant T cell numbers, infiltration or morphology and verified intact organ structures and cellular architectures (Extended Data Fig. 4d). Flow cytometric analyses confirmed similar T cell numbers between E81K-modified and control CAR T cells across different tissues including liver, lung, kidney and brain (Extended Data Fig. 4e) and liver enzymes remained unaffected in mice treated with E81K-modified 19BBz CAR T cells (Extended Data Fig. 4f,g). Lastly, serum cytokine measurements did not indicate any cytokine release syndrome-associated signatures induced by E81K-edited CAR T cells (Extended Data Fig. 4h). Taken together, there were no signs of lymphomagenesis or increased side effects associated with the improved functional persistence of E81K-modified CAR T cells.

**Extended Data Fig. 5:**
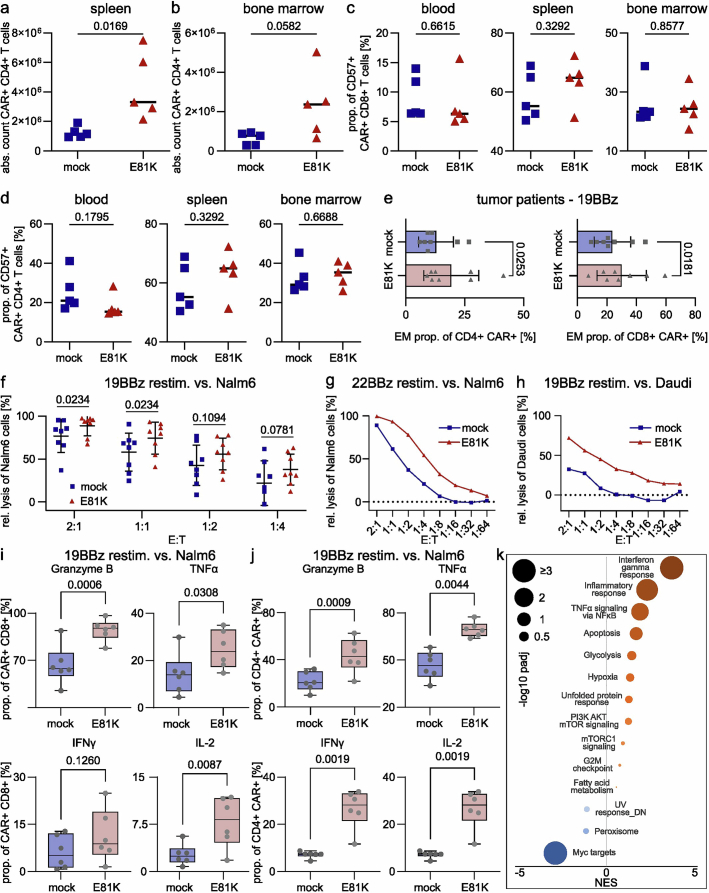
E81K-modified 19BBz CARs exhibit sustained effector function. **a**., **b**. Absolute CD4+ counts of mock and E81K-edited 19BBz CAR T cells in spleen (**a**.) and bone marrow (**b**.) of mice 16 days after CAR T cell infusion (n = 5 mice, mean; two-sided unpaired Student’s *t*-test). **c**., **d**. Flow cytometric analysis of CD57 + CD8+ (**c**.) and CD57 + CD4+ (**d**.) E81K- edited or mock 19BBz CAR T cells in blood, spleen and bone marrow of mice 16 days after CAR T cell infusion (n = 5 mice, mean; two-sided unpaired Student’s *t*-test). **e**. Percentage of effector memory cells in unstimulated CD4+ and CD8+ mock 19BBz and E81K-edited 19BBz CAR T cells generated from tumor patients (both with TRBC KO) as determined by flow cytometry (n = 8 biologically independent tumor patients, mean ± SD; two-sided paired Student’s *t*-test). **f**.-**k**. CAR T cells were stimulated two (**f**.,**i**-**k**.), or three (**g**.,**h**.) times with Nalm6 before undergoing further assays. **f**. Cytotoxic activity of mock 19BBz compared to E81K-edited 19BBz CAR T cells (both with TRBC KO) after two stimulations with Nalm6 cells using an 18 h bioluminescence assay with luciferase-expressing Nalm6 cells as targets. E:T, effector to target ratio (n = 8 biologically independent donors, mean ± SD; two-sided Wilcoxon matched-pairs signed rank test). **g**., **h**. Cytotoxic activity of mock CD22-specific BBz CAR T cells compared to E81K-edited 22BBz CAR T cells against luciferase-expressing Nalm6 tumor cells (g.) and of mock 19BBz CAR T cells compared to E81K-modified 19BBz CAR T cells against luciferase-expressing Daudi tumor cells (**h**.) using an 18 h bioluminescence assay. E:T, effector to target ratio (n = 3 technical replicates, reproduced in two biologically independent donors, mean). **i**., **j**. Flow cytometric analyses of effector cytokine and Granzyme B production of CD8+ (**i**.) and CD4+ (**j**.) E81K-edited compared to mock 19BBz CAR T cells (both with TRBC KO) after co-culture with Nalm6 cells for four hours (n = 6 biologically independent donors; two-sided paired Student’s *t*-test). Center line shows the median, box represents 25th and 75th percentiles and whiskers minimum to maximum values. **k**. GSEA of differentially expressed genes between E81K-edited and control 19BBz CAR T cells (both with TRBC KO) from one healthy donor after two Nalm6 tumor cell stimulations based on bulk RNA sequencing. Dot size represents adjusted *P*-value, permutation test. NES, normalized enrichment score.

We next assessed CAR T cell attributes 16 days after CAR injection into Nalm6-bearing mice. Mice injected with E81K-mutant CAR T cells displayed significantly higher CAR T cell numbers in peripheral blood and spleen and a similar albeit nonsignificant trend in bone marrow (Fig. 2g and Extended Data Fig. 5a,b). E81K-modified CAR T cells showed low coexpression of canonical exhaustion-associated markers PD1, LAG3 and TIM3 in vivo (Fig. 2h,i) and a similar percentage of senescence-associated CD57^+^ T cells relative to control 19BBz CAR T cells (Extended Data Fig. 5c,d), indicating that E81K does not promote T cell dysfunction in BBz CAR T cells.

Corroborating our in vitro findings from CAR T cells of healthy donors and of patients with cancer (Fig. 2b and Extended Data Fig. 5e), the E81K substitution favored an increased presence of effector memory T cells in both CD4^+^ and CD8^+^ CAR T cell subsets in vivo (Fig. 2j). To assess the role of the induced effector memory population regarding the proliferative capacity of PI3K altered or WT CAR T cell subtypes, we sorted naive, central memory, effector memory and effector T cells using flow cytometry and subjected E81K-edited and control 19BBz CAR T cells generated from each subset to repetitive antigen stimulation with Nalm6 cells. We found that E81K-mutant effector memory CAR T cells outperformed all other T cell subsets and each subset of unmodified 19BBz CARs, validating their superior antigen-dependent expansion potential (Fig. 2k).

## E81K substitution affords enhanced functional persistence of BBz CAR T cells in vivo

**Extended Data Fig. 6:**
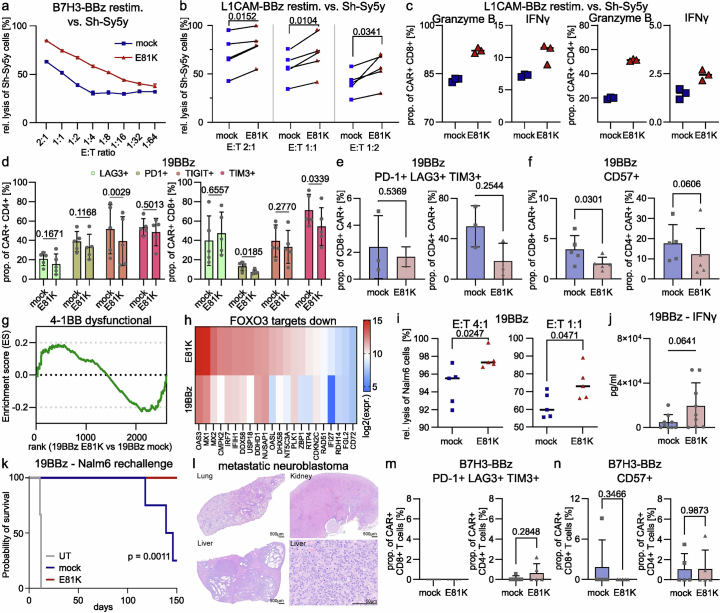
E81K-mutated 19BBz CAR T cells preserve low expression of senescence- and exhaustion-associated markers. **a**. Cytotoxic activity of B7H3-specific BBz CAR T cells compared to E81K-edited B7H3-BBz CAR T cells at the fourth antigen stimulation using an 18 h bioluminescence assay with luciferase-expressing Sh-Sy5y cells as targets. E:T, effector to target ratio (mean of n = 3 technical replicates, reproduced in two biologically independent donors). **b**. Cytotoxic activity of mock L1CAM-BBz compared to E81K-edited L1CAM-BBz CAR T cells (both with TRBC KO) at the fourth antigen stimulation with luciferase-expressing Sh-Sy5y cells as target cells using an 18 h bioluminescence assay. E:T, effector to target ratio (n = 5 biologically independent donors; two-sided paired Student’s *t*-test). **c**. Granzyme B and IFNγ production of CD8+ (left panel) and CD4+ (right panel) E81K-edited compared to mock L1CAM-specific BBz CAR T cells (both with TRBC KO) that were exposed to three repeated Sh-Sy5y stimulations and co-cultured with Sh-Sy5y cells for 16 h, measured by flow cytometry (mean of n = 3 technical replicates, reproduced in two biologically independent donors). **d**. Flow cytometric analysis of the exhaustion markers LAG3, PD-1, TIGIT and TIM3 in CD4+ (left) and CD8+ (right) E81K-edited or mock 19BBz CAR T cells after three antigen stimulations (both with TRBC KO) (n = 5 biologically independent donors; two-sided paired t-test; mean + SD). **e**. Co-expression of exhaustion markers LAG3, PD-1, and TIM3 on CD8+ (left) and CD4+ (right) E81K-edited or mock 19BBz CAR T cells (both with TRBC KO) generated from tumor patients after three rounds of antigen stimulations as determined by flow cytometry (n = 3 biologically independent donors; mean + SD; two-sided paired Student’s *t*-test). **f**. CD57 expression on CD8+ (left) and CD4+ (right) E81K- edited or mock 19BBz CAR T cells after three antigen stimulations with Nalm6 (n = 5 biologically independent donors; mean + SD, two-sided paired Student’s *t*-test). **g**. GSEA using the “4-1BB dysfunctional” gene signature^42^ comparing E81K-mutated versus *PIK3CD* wild-type (WT, “mock”) 19BBz CAR T cells from one healthy donor after two antigen stimulations (both with TRBC KO). **h**. Heatmap showing genes upregulated upon *FOXO3* KO^41^, highlighting enrichment of this gene set in E81K-edited compared to mock 19BBz CAR T cells from one healthy donor (both with TRBC KO) based on bulk RNA sequencing. *FOXO3* KO has been associated with reversal of dysfunction in 4-1BB based CAR T cells^42^. **i**. *Ex vivo* cytotoxic activity of mock 19BBz and E81K-edited 19BBz CAR T cells isolated from the spleens of mice 16 days after CAR T cell injection determined by an 18 h bioluminescence assay with luciferase-expressing Nalm6 cells as targets. Effector to target, E:T ratio (n = 5 mice, mean; two-sided unpaired Student’s *t*-test). **j**. IFNγ serum levels of mice treated with E81K-edited or control 19BBz CAR T cells 16-18 days after T cell infusion as measured by multiplexed ELISA (n = 9 mice; mean ± SD; two-sided unpaired Student’s *t*-test; samples were also used for Extended Data Fig. 4h). **k**. In vivo rechallenge experiment with Nalm6 cells to evaluate functional persistence of 19BBz TRBC KO CAR T cells with or without E81K mutation. Survival data corresponds to the cohort described in Fig. 3a-b. (n = 4 mice, two-sided Mantel-Cox test), UT, untransduced. **l**. Representative for n = 3 H&E stainings from lung, kidney, and liver 28 days after infusion of untransduced T cells in Sh-Sy5y-bearing NSG mice (scale bar size as indicated). **m**.-**n**. Flow cytometric analysis of TIM3, PD-1 and LAG3 triple positive (**m**.) and CD57+ (**n**.) TRBC KO B7H3-BBz CAR T cells with and without E81K mutation for CD8+ (left) and CD4+ (right) T cells 10 days after infusion into Sh-Sy5y-bearing mice (n = 5 mice, mean ± SD; two-sided unpaired Student’s *t*-test).

To assess the long-term functionality of E81K-modified BBz CAR T cells, we performed repetitive stimulations with hematological or solid tumors and found that E81K-mutant CAR T cells retained their robust cytotoxic advantage and sustained higher levels of effector cytokine responses even after several rounds of tumor exposure (Extended Data Figs. 5f–k and 6a–c). Consistent with the sustained functionality, E81K-edited CAR T cells derived from healthy donors or patients with cancer exhibited no increased expression of exhaustion or senescence markers after repetitive antigen stimulations (Extended Data Figs. 6d–f). Profiling of transcriptional changes confirmed the heightened effector state of E81K-edited BBz CAR T cells (Extended Data Fig. 5k) and indicated reduced T cell dysfunction mediated by the transcription factor FOXO3 (ref. ^41^) (Extended Data Fig. 6g,h), a main driver of 4-1BB-based CAR T cell impairment^42^.

To delineate whether in vivo persisting E81K-edited BBz CAR T cells maintain effective antitumor responses, we isolated CAR T cells from the spleen of treated mice and reexposed them to Nalm6 cells ex vivo. Strikingly, the retrieved E81K-mutant 19BBz CAR T cells outperformed control CAR T cells in their cytolytic capacities (Extended Data Fig. 6i). The observations of increased antitumor activity of persisting E81K-modified CAR T cells were concordant with enhanced IFNγ levels in the blood of mice 16–18 days after treatment with E81K-edited BBz CAR T cells (Extended Data Fig. 6j).

**Fig. 3:**
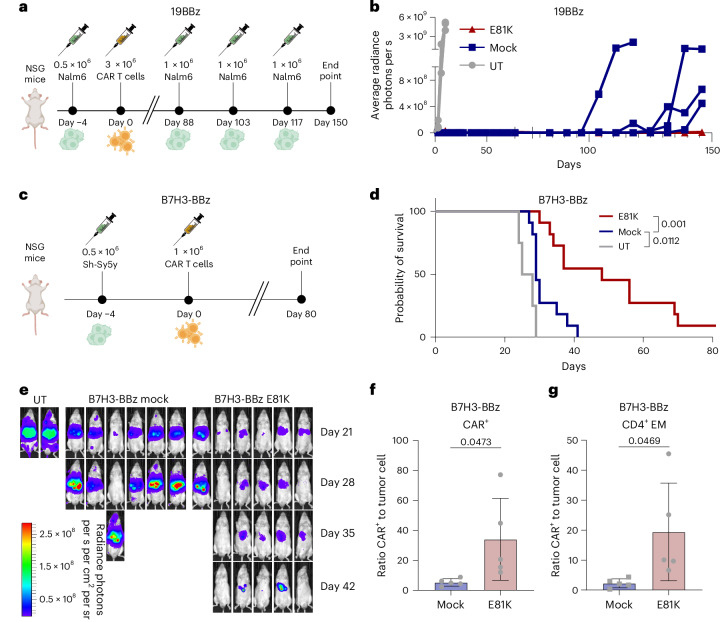
The E81K edit induces functional persistence of BBz CAR T cells and improves tumor control in vivo*.* **a**,**b**, In vivo tumor rechallenge experiment with Nalm6 cells to evaluate functional persistence of 19BBz *TRBC*-KO CAR T cells with or without E81K substitution. **a**, Experimental timeline. **b**, Tumor progression was assessed by bioluminescence imaging (*n* = 4 mice per group). **c**–**e**, Luciferase-expressing Sh-Sy5y cells were i.v. injected into NSG mice followed by i.v. administration of UT T cells or B7H3-BBz *TRBC*-KO CAR T cells with or without E81K substitution. **c**, Experimental timeline. **d**, Survival of the cohort (E81K B7H3-BBz: *n* = 11, control B7H3-BBz: *n* = 11 mice per group, UT: *n* = 4 mice per group; T cells from two biologically unrelated healthy donors; two-sided Mantel–Cox test). **e**, Bioluminescence imaging of Sh-Sy5y-bearing mice (representative of **d**) treated with UT T cells, B7H3-BBz *TRBC*-KO (mock) and E81K-edited B7H3-BBz *TRBC*-KO CAR T cells at the indicated time points (ventral view). **f**,**g**, Data from the liver of mice (*n* = 5) treated as shown in **c** and collected 10 days after injection of either B7H3-BBz or E81K-modified B7H3-BBz CAR T cells (with *TRBC* KO). **f**, CAR T cell-to-tumor cell ratio (mean ± s.d.; two-sided unpaired Student’s *t*-test). **g**, Effector memory CD4^+^ CAR T cell-to-tumor cell ratio (mean ± s.d.; two-sided unpaired Student’s *t*-test).

To confirm the improved functional persistence of E81K-edited CAR T cells, we used a model of tumor relapse after initial response, which remains a major challenge in CAR T cell therapy^43^. To mimic this clinical scenario, we selected a setting in which both E81K-edited and control 19BBz CAR T cells induced full remission after initial CAR T cell infusion and rechallenged the mice with three consecutive rounds of Nalm6 injections at late time points (days 88, 103 and 117 after initial Nalm6 injection) (Fig. 3a). While all mice harboring control CAR T cells developed tumor relapses shortly after Nalm6 rechallenge, all E81K-mutant 19BBz CAR T cells were able to control tumor rechallenges (Fig. 3b), resulting in significantly improved survival (Extended Data Fig. 6k). Together, these data show that E81K-mutant CAR T cells retain their functional capacity long-term, thereby outperforming prototypic BBz CAR T cells.

## E81K base edit improves BBz CAR T cell responses against solid tumors

Given the great challenge in enhancing CAR T cell efficacy against solid tumors, we evaluated B7H3-BBz CAR T cells with or without the E81K substitution at stress test doses in a xenograft model of metastatic Sh-Sy5y neuroblastoma endogenously expressing B7H3/CD276 (ref. ^44^) (Fig. 3c and Extended Data Fig. 6l). E81K-modified B7H3-BBz CAR T cells demonstrated superior tumor control, resulting in significantly prolonged survival of treated mice (Fig. 3d,e). This therapeutic benefit was associated with an augmented CAR T cell-to-tumor ratio in the tumor-bearing liver (Fig. 3f). Consistent with our findings in hematological tumors, E81K-modified CAR T cells favored the presence of effector memory cells in the liver, leading to a higher effector memory CAR T cell-to-tumor cell ratio (Fig. 3g) while preventing elevated expression of exhaustion or senescence markers in vivo (Extended Data Fig. 6m,n).

In summary, the E81K substitution enhances the functional persistence of BBz CAR T cells without increasing safety concerns, reinforcing their high therapeutic potential.

## E81K editing enhances metabolic fitness in BBz CAR T cells

**Fig. 4:**
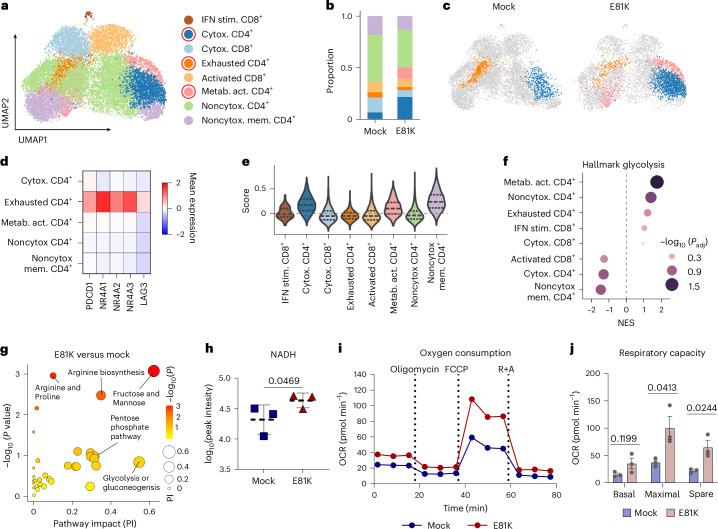
Increased PI3K activity in E81K-edited 19BBz CAR T cells is associated with enhanced effector function and metabolic fitness. Unmodified (mock) or E81K-modified 19BBz CAR T cells (*TRBC* KO) from one healthy donor were sorted after two antigen stimulations with Nalm6 tumor cells and subsequently subjected to scRNA-seq analysis. **a**, UMAP embedding of all analyzed cells. Clusters of interest are encircled in red. **b**, Cluster distribution within each condition. **c**, Comparison of selected populations of interest for E81K-modified and unmodified 19BBz CAR T cells as shown by UMAP embedding. **d**, Level of expression of the indicated exhaustion-related genes across all CD4^+^ clusters. **e**, The score for genes upregulated in CD19-targeting compared to exhaustion-prone HA-targeting CD4 effector memory CAR T cells^45^ for all identified clusters. **f**, GSEA of the MSigDB Hallmark glycolysis gene set was performed for all identified clusters (permutation test). **g**, Joint transcriptomic and metabolomic pathway analysis (using MetaboAnalyst; www.metaboanalyst.ca) showing upregulated metabolic pathways in E81K-modified 19BBz CAR T cells relative to controls after two stimulations with Nalm6 target cells. Top-ranked pathways are labeled. Transcriptomic data were used from experiment shown in **a**,**b**. Metabolic analysis is shown in Extended Data Fig. 8e–g (hypergeometric testing). **h**, Cellular ion counts of NADH across E81K-modified and 19BBz control CAR T cells after second Nalm6 tumor stimulation measured by mass spectrometry (*n* = 3 biologically independent healthy donors; mean ± s.d.; two-sided paired Student’s *t*-test). **i**, OCR traces as measured by seahorse mitochondrial stress test for *TRBC*-KO control 19BBz (mock) and E81K-edited 19BBz CAR T cells after exposure to three repeated stimulations with Nalm6 cells. Lines mark the addition of oligomycin (2 μM), FCCP (1 μM) and rotenone + antimycin A (R + A; 0.5 μM) (mean of *n* = 5 technical replicates for one representative T cell donor of three donors). **j**, Average basal OCR, maximal OCR and spare respiratory capacity levels in seahorse mitochondrial stress test (*n* = 3 biologically independent T cell donors; mean ± s.d.; two-sided paired Student’s *t*-test).

**Extended Data Fig. 7:**
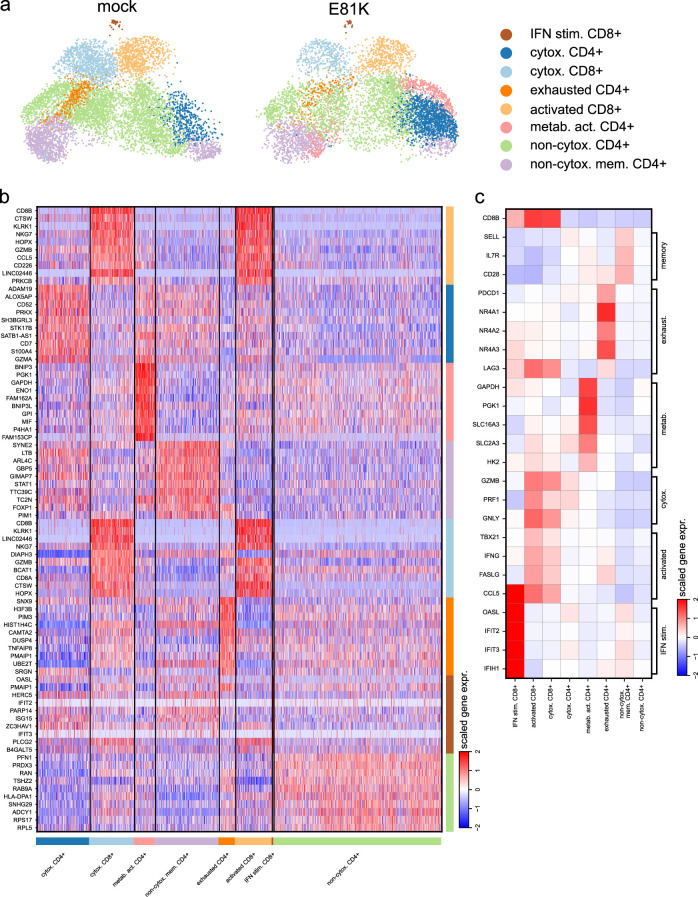
Single-cell RNA sequencing reveals distinct cell populations in E81K-modified BBz CAR T cells. Unmodified (“mock”) or E81K-modified (“E81K”) 19BBz CAR T cells (both with TRBC KO) from one healthy donor were sorted after two antigen stimulations with Nalm6 tumor cells and subsequently subjected to scRNAseq analysis. **a**. UMAP embedding of all unmodified 19BBz (“mock”) and E81K-modified 19BBz (“E81K”) CAR T cells. **b**. Heatmap of the top 10 differentially expressed genes for the identified clusters. **c**. Heatmap showing average expression of marker genes defining the eight identified clusters.

To identify underlying mechanisms resulting in improved functional persistence of E81K-mutant BBz CAR T cells, we performed single-cell RNA sequencing (scRNA-seq) on sorted antigen-stimulated 19BBz CAR T cells with and without E81K substitution. Unsupervised clustering was followed by integration into *n* = 8 transcriptionally distinct clusters (Supplementary Tables 1–8) on the basis of selected marker gene expression. While we observed that CD4^+^ T cells are generally enriched in the E81K context, we identified two clusters of particular relevance when comparing the two conditions (Fig. 4a–c and Extended Data Fig. 7): a cytotoxic CD4^+^ T cell cluster enriched in E81K-modified CAR T cells and a metabolically active CD4^+^ cluster uniquely enriched in E81K-modified 19BBz CAR T cells but nearly absent in their unmodified counterparts. Moreover, E81K-modified 19BBz CAR T cells were depleted in a CD4^+^ cluster with high expression of exhaustion-related genes *NR4A1*, *NR4A2*, *NR4A3*, *PDCD1* and *LAG3*, indicative of an exhaustion-like phenotype (Fig. 4a–d). Consistent with these findings, gene set enrichment analysis (GSEA) comparing CD19 CAR T cells to exhaustion-prone HA CAR T cells^45^ revealed transcriptional similarities of the exhaustion-like CD4⁺ T cell cluster to HA CAR T cells. In contrast, the CD4⁺ clusters enriched in E81K-modified 19BBz CAR T cells more closely correlated with the transcriptional profile of functional CD19 CAR T cells (Fig. 4e).

**Extended Data Fig. 8:**
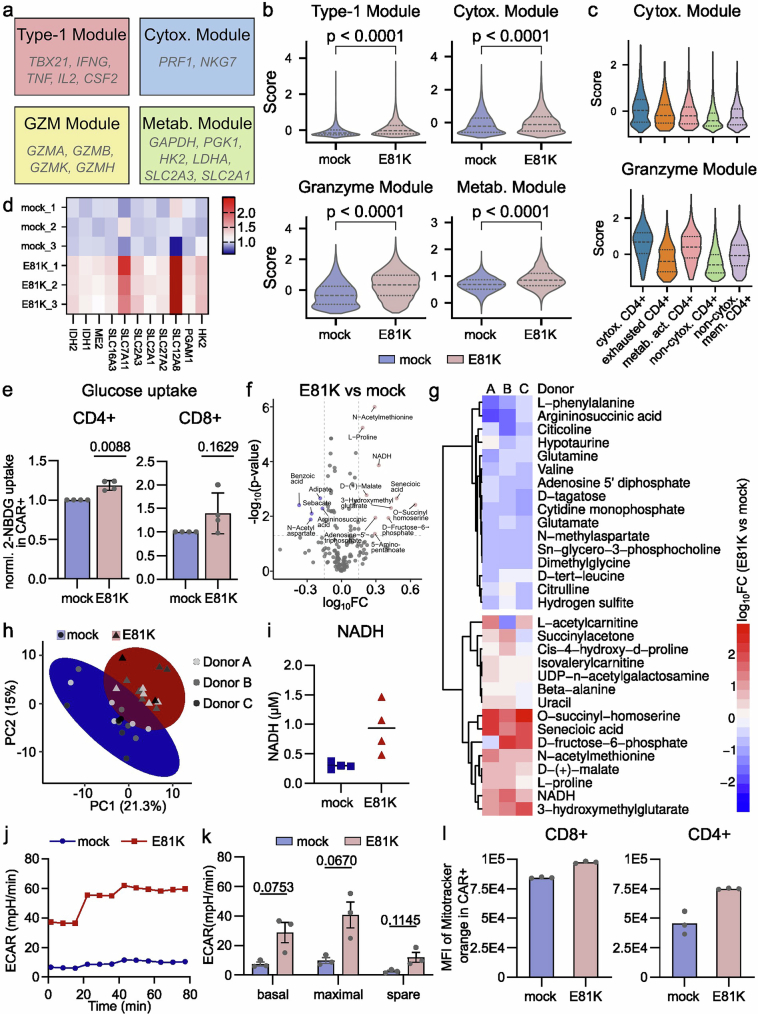
E81K editing increases metabolic fitness in BBz CAR T cells. **a**.-**c**. Comparison of gene modules defined by expression of distinct genes (**a**.) for mock 19BBz versus E81K-modified 19BBz CAR T cells from one healthy donor (**b**.) and for all CD4+ clusters (**c**.) determined by scRNA sequencing. Statistical analyses are performed using two-sided unpaired Student’s t-test. Metab. = metabolic, Cytox. = cytotoxic. **d**. Heatmap demonstrating expression of glycolysis-associated genes in TRBC KO E81K-edited and TRBC KO mock 19BBz CAR T cells after two exposures to Nalm6 target cells based on bulk RNA sequencing (n = 3 technical replicates per group from one healthy donor); rows normalized to mean of control group. **e**. 2-NBDG uptake of antigen-stimulated CD8+ mock 19BBz and E81K-edited 19BBz CAR T cells (both with TRBC KO) as analyzed by flow cytometry (n = 4 biologically independent donors, values were norml. to mock control of each donor; mean ± SD; one-sample t-test). **f**. Volcano plot of metabolites in E81K-edited compared to mock 19BBz CAR T cells for three independent donors. (Two-sided unpaired Student’s *t*-test; Cut offs were log₁₀(Fold Change(FC)) > 0.15 or < 0.15 and p-value < 0.05.) **g**. Heatmap of log₁₀(Fold Change (FC)) of metabolites in E81K-edited compared to mock 19BBz CAR T cells for each independent donor. The heatmap displays log₁₀(FC), color-coded to indicate magnitude and direction of change. (n = 3 biologically independent donors, two-sided unpaired Student’s *t*-test; only metabolites with a p-value < 0.01 in at least one donor are shown.) **h**. Donor-corrected Principal Component Analysis (PCA) of PC1 and PC2 from metabolite profiles of mock 19BBz and E81K-modified 19BBz CAR T cells (both with TRBC KO) from n = 3 biologically independent donors 24 h after second Nalm6 tumor cell stimulation. **i**. Absolute NADH levels of mock 19BBz or E81K-modified 19BBz CAR T cells (both with TRBC KO) 24 h after second Nalm6 tumor cell stimulation (n = 4 replicates per group, representative of n = 2 biologically independent donors). **j**. Extracellular acidification rate (ECAR) traces as measured by seahorse mitochondrial stress test for mock 19BBz and E81K-edited 19BBz CAR T cells (both with TRBC KO) after exposure to three repeated stimulations with Nalm6 cells (mean of n = 5 technical replicates for one donor, representative for n = 3 biologically independent donors). **k**. Average basal ECAR, maximal ECAR, and spare glycolytic capacity levels in seahorse mitochondrial stress test (n = 3 biologically independent donors, mean ± SD, two-sided paired Student’s *t*-test). **l**. Mean fluorescent intensity (MFI) of mitochondrial mass content in CD8+ (left panel) and CD4+ (right panel) mock 19BBz and E81K-edited 19BBz CAR T cells after co-culture with Nalm6 cells following two consecutive rounds of cancer target stimulations as detected by MitoTracker orange staining (n = 3 technical replicates, reproduced in n = 2 biologically independent donors).

Concordant with our findings of increased effector function, E81K-modified CAR T cells displayed enhanced cytotoxic signatures characterized by high expression of effector genes including granzymes and perforin, and showed a specific enrichment of cytotoxic CD4^+^ T cells (Extended Data Fig. 8a–c)^46^.

The metabolically active CD4⁺ T cell cluster highly enriched in E81K-modified 19BBz CAR T cells displayed increased glycolytic activity (Fig. 4f). These findings were supported by an overall heightened expression of glycolysis-related genes and increased glucose uptake in E81K-modified 19BBz CAR T cells (Extended Data Fig. 8a,b,d,e). Parallel metabolomic and transcriptomic analyses revealed altered intracellular metabolite levels in E81K-edited CAR T cells with a pronounced increase in NADH levels (Fig. 4g,h and Extended Data Fig. 8f–i). Together, these findings indicate an overall favorable metabolic capacity induced by the E81K substitution in BBz CAR T cells (Fig. 4g,h and Extended Data Fig. 8a,b,d–i).

Considering the pivotal role of metabolic fitness in sustaining long-term T cell functionality, we conducted a real-time metabolic flux analysis of E81K-edited versus mock CAR T cells following repeated tumor antigen exposure. E81K-mutant CAR T cells showed higher basal and maximal oxygen consumption, a significantly increased spare respiratory capacity (Fig. 4i,j) and a higher spare glycolytic capacity (Extended Data Fig. 8j,k). This improved CAR T cell fitness was accompanied by an increased mitochondrial mass of E81K-mutant CAR T cells relative to their unedited counterparts (Extended Data Fig. 8l). Taken together, transcriptional and metabolic analyses indicate that the E81K substitution enhances cytolytic capacity and metabolic fitness of BBz CAR T cells while mitigating T cell exhaustion.

## Attenuated PI3K signaling improves 28z-based CAR T cells

**Extended Data Fig. 9:**
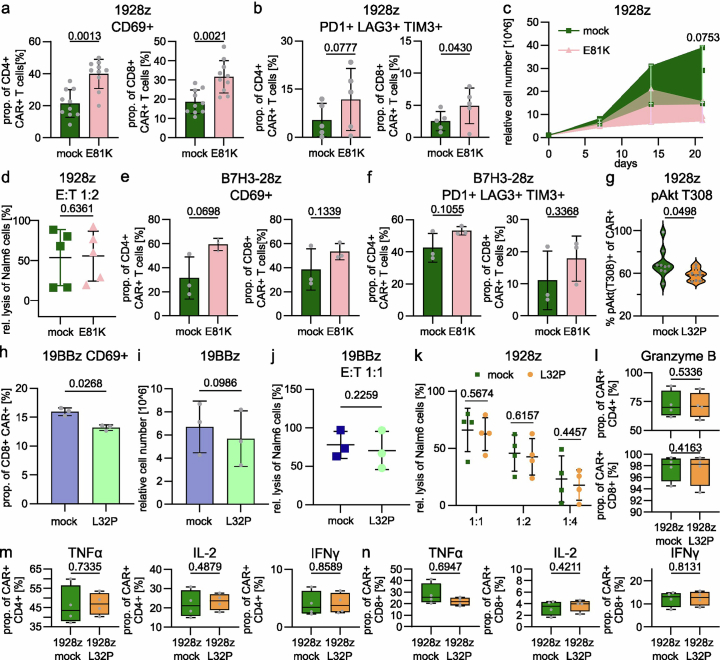
Differential signaling strength induced by individual point mutations shapes CAR T cell phenotypes in a co-stimulatory dependent manner. **a**. CD69 expression levels in CD4+ (left) and CD8+ (right) 1928z as compared to E81K-modified 1928z determined by flow cytometry without further antigen stimulation (n = 10 biologically independent donors, mean ± SD; two-sided paired Student’s *t*-test; samples for mock 1928z partially overlap with data presented in Fig. 5a). **b**. Co-expression of exhaustion markers LAG3, PD-1, and TIM3 on CD4+ (left) and CD8+ (right) E81K-edited or control 1928z CAR T cells after three rounds of antigen stimulations as determined by flow cytometry (n = 5 biologically independent donors; mean + SD; two-sided paired Student’s *t*-test). **c**. Antigen-dependent cell growth of mock 1928z and E81K-edited 1928z CAR T cells (both with TRBC KO) during n = 3 weekly stimulations with Nalm6 cells at an effector to target (E:T) ratio of 2:1 (n = 3 biologically independent donors; mean± SD; two-sided paired Student’s t-test). **d**. Cytotoxic activity of mock 1928z compared to E81K-edited 1928z CAR T cells against Nalm6 tumor cells using an 18 h bioluminescence assay at indicated effector to target ratio (n = 5 biologically independent donors, mean ± SD, two-sided paired Student’s *t*-test). **e**. CD69 expression levels in CD4+ (left) and CD8+ (right) B7H3-28z as compared to E81K-modified B7H3-28z determined by flow cytometry without further antigen stimulation (n = 3 biologically independent donors, mean ± SD; two-sided paired Student’s *t*-test). **f**. Co-expression of exhaustion markers LAG3, PD-1, and TIM3 on CD4+ (left) and CD8+ (right) E81K-edited or mock B7H3-28z CAR T cells after one round of antigen stimulation as determined by flow cytometry (n = 4 biologically independent donors; mean + SD; two-sided Wilcoxon matched-pairs signed rank test). **g**. Flow cytometry-based analysis of phosphorylated (p)-AKT T308 in mock 1928z and L32P-edited 1928z CAR T cells (“L32P”) after 1 h of stimulation with Nalm6 at an effector to target (E:T) ratio of 1:1. Data are shown in triplicates from n = 3 biologically independent donors; two-sided unpaired Student’s *t*-test. **h**. CD69 expression levels in mock 19BBz as compared to L32P-modified 19BBz CAR T cells 72 h after stimulation with Nalm6 cells as determined by flow cytometry (n = 3 biologically independent donors, mean ± SD; two-sided paired Student’s *t*-test). **i**. Antigen-dependent cell growth of mock 19BBz and L32P-edited 19BBz CAR T cells after stimulation with Nalm6 cells at an effector to target (E:T) ratio of 2:1 (n = 3 biologically independent donors, mean ± SD; two-sided paired Student’s *t*-test). **j**. Cytotoxic activity of mock 19BBz compared to L32P-edited 19BBz CAR T cells against luciferase-expressing Nalm6 cells at an E:T ratio of 1:1 (n = 3 biologically independent donors, mean ± SD; two-sided paired Student’s *t*-test). **k**. Cytotoxic activity of mock 1928z compared to L32P-edited 1928z CAR T cells using an 18 h bioluminescence assay with luciferase-expressing Nalm6 cells as targets for indicated effector to target ratios (n = 4 biologically independent donors, mean ± SD; two-sided paired Student’s *t*-test). **l**.-**n** Granzyme B production in CD4+ and CD8+ (**l**.) and effector cytokine production in CD4+ (**m**.) and CD8+ (**n**.) L32P-edited and mock 1928z CAR T cells 4 h after stimulation with Nalm6 cells as measured by flow cytometry (n = 4 biologically independent donors; two-sided paired Student’s *t*-test). Center line shows the median, box represents 25th and 75th percentiles and whiskers minimum to maximum values.

Given the beneficial properties conferred upon BBz CAR T cells by the E81K substitution, we next sought to assess its role in context of the 28z CAR design. Similar to our findings in BBz CAR T cells, induction of E81K in 1928z CARs boosted CD69 expression relative to unedited controls, reinforcing mutation-induced changes in T cell activation levels (Extended Data Fig. 9a). However, E81K editing in 28z CAR T cells was associated with augmented expression of exhaustion-related markers (Extended Data Fig. 9b) and the inability to enhance proliferative capacity and cytolytic properties (Extended Data Fig. 9c,d). We observed similar albeit not statistically significant trends in T cells expressing high tonic-signaling B7H3-specific CARs following introduction of the E81K alteration (Extended Data Fig. 9e,f). Together, these results demonstrate that the E81K substitution confers functional benefits specifically in the context of 4-1BB-based CARs but not in the CD28 costimulation-based CAR framework, underscoring the need for signaling modulation tailored to the respective CAR design.

**Fig. 5:**
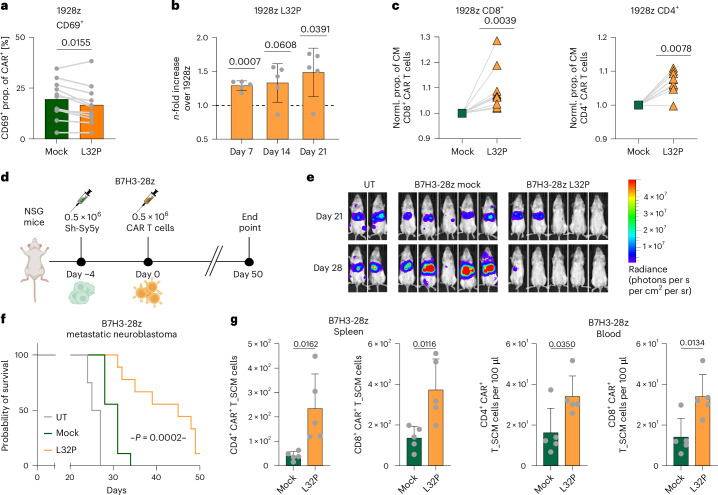
The L32P substitution enhances antitumor efficacy of 28z-based CAR T cells in vivo by increasing memory formation. **a**, CD69 expression levels in mock 1928z as compared to L32P-modified 1928z CAR T cells determined by flow cytometry without further antigen stimulation (*n* = 11 biologically independent donors, mean ± s.d.; two-sided paired Student’s *t*-test). **b**, *n*-fold CAR T cell expansion for L32P-edited relative to unmodified 1928z CAR T cells 7 days after one (day 7), two (day 14) and three (day 21) stimulation(s) with Nalm6 cells (two-sided one-sample *t*-test; *n* = 5 biologically independent donors, mean ± s.d.). **c**, Fold enrichment of the central memory (CD62L^+^CD45RA^−^) population in L32P-edited 1928z relative to mock 1928z CAR T cells within the CD8^+^ (left) and CD4^+^ (right) CAR^+^ compartment. Statistical analysis was performed using a two-sided one-sample Wilcoxon test (*n* = 9 biologically independent donors). **d**–**f**, Luciferase-expressing Sh-Sy5y cells were i.v. injected into NSG mice followed by i.v. administration of UT T cells or B7H3-28z CAR T cells with or without L32P substitution. **d**, Experimental timeline. **e**, Bioluminescence imaging of Sh-Sy5y-bearing mice (representative of **f**) treated with UT, mock B7H3-28z and L32P-edited B7H3-28z CAR T cells at the indicated time points (ventral view). **f**, Survival of the cohort (L32P B7H3-28z: *n* = 9, mock B7H3-28z: *n* = 9 mice, UT: *n* = 4 mice per group; T cells from two biologically unrelated healthy donors; two-sided Mantel–Cox test). **g**, Data from the liver of mice (*n* = 5) treated as shown in (**d**) and collected 10 days after injection of either mock B7H3-28z or L32P-modified B7H3-28z CAR T cells. Absolute count of SCM CD4^+^ and CD8^+^ CAR T cells in spleen and blood (*n* = 5 mice per group, mean ± s.d.; two-sided unpaired Student’s *t*-test).

Therefore, we selected the top hit (L32P) in 28z CAR T cells on the basis of our initial screening results and investigated the phenotypical and functional consequences resulting from this substitution. In accordance with attenuated PI3K signaling mediated by the L32P substitution (Fig. 1i,j and Extended Data Fig. 9g), we observed decreased levels of T cell activation in unstimulated L32P-edited 1928z and antigen-stimulated L32P-edited CD8^+^ 19BBz CAR T cells (Fig. 5a and Extended Data Fig. 9h). In contrast to 19BBz CAR T cells that did not benefit from the L32P substitution (Extended Data Fig. 9i,j), L32P-mutant 1928z CAR T cells showed considerably increased antigen-dependent proliferation potential and retained the high effector function attributed to the 28z CAR design (Fig. 5b and Extended Data Fig. 9k–n). In line with these observations, L32P-mutant 1928z CARs exhibited a less differentiated T cell state as indicated by an increased proportion of central memory T cells compared to mock-edited 1928z CAR T cells (Fig. 5c).

We next evaluated L32P-edited B7H3-28z CAR T cells in the challenging context of a solid tumor model in vivo. We found that L32P-mutant B7H3-28z CAR T cells significantly improved overall survival of mice in a metastatic neuroblastoma model compared to B7H3-28z controls (Fig. 5d–f). Consistent with our previous findings, the enhanced tumor control was associated with a less differentiated T cell phenotype, as indicated by a significantly increased stem-cell-like memory (SCM) T cell pool in L32P-edited compared to mock B7H3-28z CAR T cells on day 10 after injection (Fig. 5g).

Overall, these findings underscore the distinct role of PI3K–AKT signaling in BBz and 28z CAR T cell biology and highlight both the technological and the biological advances of our ROADSTAR approach. Targeted induction of point mutations by base editing enables precise calibration of signaling strength for each CAR design, enabling refined cell products with enhanced therapeutic potency.

## Discussion

Herein, we develop ROADSTAR to fine-tune endogenous PI3K–AKT signaling in CAR T cells through introduction of targeted point mutations. This base-editing screening platform revealed distinct point mutations in the regulatory ABD of p110δ that yield calibrated signaling activities tailored to either 28z-based or BBz-based CAR designs, resulting in favorable phenotypic, metabolic and functional T cell properties.

BBz CAR T cells showed highest enrichment for the PI3K-activating E81K substitution. Intriguingly, this substitution has been reported in persons with activated PI3Kδ syndrome, an autosomal dominant immune deficiency caused by an increased activity of the PI3K–AKT signaling pathway in immune cells^19,47–49^. While these clinical data bolster our findings of greater PI3K–AKT pathway activity induced by E81K, we found that the functional consequences for T cells are contrasting in the context of BBz CAR T cells. Activating PI3Kδ germline alterations foster early T cell differentiation at the expense of memory formation, which ultimately drives T cell exhaustion and senescence, thereby impairing immune responses^19,47,48,50^. In contrast, E81K-mutant BBz CAR T cells demonstrated enhanced antitumor activity upon repeated antigen exposures across different tumor entities in vitro and improved therapeutic efficacy against established hematological and solid tumors in vivo without driving T cell dysfunction.

Thus, the increased PI3K–AKT signaling activity in E81K-modified CAR T cells resulted in enhanced effector function while preserving the beneficial memory features ascribed to this CAR design. This suggests that 4-1BB costimulation incorporated in the CAR design promotes memory formation in a nonredundant way to PI3K signaling.

Mechanistically, metabolic and single-cell transcriptional profiling revealed improved metabolic capacities of E81K-edited BBz CAR T cells associated with increased spare respiratory capacity and mitochondrial fitness after repetitive antigen exposures—features that have been linked to improved T cell functionality^28,46,51^.

The additional signaling capacity conferred by the E81K substitution was not beneficial in the context of 28z CAR T cells. Instead, the L32P substitution identified as the top hit in 28z CAR T cells resulted in diminished PI3K–AKT signaling capacity, thereby limiting early T cell differentiation and activation profiles in 28z CAR T cells while simultaneously enhancing their proliferative capacity. These desirable attributes of L32P-mutant 28z CAR T cells culminated in superior control of a metastatic solid tumor in vivo.

One concern of activating *PIK3CD* point mutations is the potential of malignant transformation. Among over 67,000 individuals reported in cBioPortal, no cases of T cell leukemias harboring L32P or E81K substitutions have been reported. Likewise, there are no described T cell leukemias arising in persons with E81K substitutions, suggesting safety advantages compared to CRISPR–Cas-mediated KO of tumor suppressor genes or overexpression of mutations that have been implicated in T cell malignancies^52,53^. In accordance, our long-term in vivo experiments did not reveal any signs of toxicities or malignant transformation in mice treated with E81K-edited BBz CAR T cells.

This work highlights ROADSTAR as a strategy to modulate T cell intrinsic signaling pathway activity by targeted base edits, enabling differential signaling capacities tailored to the specific CAR design. This strategy differs from previous work focused on changes in the CAR construct itself and provides a notable advantage compared to genetic KO or exogenous overexpression by enabling precise calibration of signaling activation levels and preserving positive and negative feedback regulation^15,17,25,53,54^. Notably, our study emphasizes the importance of the underlying T cell biology imparted by the relevant CAR design in determining the beneficial nature of a point mutation to unleash full therapeutic potential. Previous studies identified *PIK3CD* variants capable of modulating T cell activity through base-editing screens in untransduced T cells^21,55^. However, such approaches that do not account for CAR design-specific signaling effects might select for highly activating mutations that, when combined with additional signaling provided in a CAR context, could risk inducing T cell overstimulation, hyperproliferation or dysfunction.

Overall, ROADSTAR enabled the identification of distinct point mutations induced by base editing that confer functional advantage and favorable T cell profiles to 28z and BBz CARs by calibrating endogenous signaling strength. These insights pave the way for more effective cellular cancer immunotherapies while simultaneously advancing our understanding of CAR T cell biology.

## Methods

### Cell lines and culture conditions

Cell lines used were luciferase-expressing and GFP-expressing Nalm6 (M.S., Columbia University), Kelly, SK-N-AS and Sh-Sy5y (University Children’s Hospital Tübingen) and Daudi (kindly provided by D. Sonanini, University Hospital Tübingen). Cells were PCR-tested for *Mycoplasma* and found to be negative. SUP-T1 (S. Hailfinger, University Hospital Münster), Sh-Sy5y, Kelly and Daudi cells were cultured in RPMI-1640 (Gibco) with L-glutamine, 10% FBS and 1% penicillin–streptomycin. Nalm6 cells were additionally supplemented with 10 mM HEPES, 1× nonessential amino acids (NEAA), 1 mM sodium pyruvate and 50 μM β-mercaptoethanol (all Gibco). SK-N-AS were cultured in DMEM (Gibco) supplemented with L-glutamine, 10% FBS, 1% penicillin–streptomycin and 1× NEAA.

### Isolation, activation and expansion of primary T cells

Fresh blood products from healthy deidentified volunteers were obtained from Zentrum für klinische Transfusionsmedizin, Tübingen. Samples from pretreated participants with cancer were isolated from 60–81-year-old individuals suffering from different cancer entities including sarcoma, lung cancer and breast cancer after several rounds of chemotherapy. Samples from pretreated participants with tumors were obtained from the Medical Department, University Hospital Tübingen. All blood samples were handled following the ethical and safety procedures approved by the ethic commission of the University of Tübingen (783/2023BO2, 846/2020BO2). Informed consent was obtained from the participants. All samples of human origin were destroyed after the analysis. Peripheral blood mononuclear cells were isolated from blood products and T cells were subsequently isolated using human Pan T cell isolation kit (Miltenyi Biotec, 130-096-5359). T cells were activated at a cell-to-bead-ratio of 1:1 with human T activator CD3/CD28 Dynabeads (Thermo Fisher, 11161D) in the presence of 5 ng ml^−1^ IL-7 and IL-15 (Miltenyi Biotec, 130-095-363 and 130-095-765). Cells were cultured at 1 × 10^6^ cells per ml in X-Vivo15 (Lonza, BEBP02-061Q), which was supplemented with 5% human AB-positive serum and 1% penicillin–streptomycin.

### Base editor mRNA in vitro transcription (IVT)

pCMV-ABEmax (Addgene, 112094) and pCMV-AncBE4max (Addgenes, 112095) were used as templates. IVT was performed using the T7MEGAscript kit (Thermo Fisher, AMB13345). The plasmids were linearized through AgeI-HF, followed by IVT according to manufacturer’s instructions. Transcripts were capped with the CleanCapAG (Tebubio, N-7113) and subjected to poly(A) tailing (Thermo Fisher, AM1350) according to the manufacturer’s instructions. mRNA was purified through overnight LiCl precipitation and mRNA was stored at −150 °C.

### Retroviral production and pooled base-editing screen in human primary CAR T cells

Base-editing sgRNAs (Supplementary Tables 9 and 10) were cloned into the previously described SFGγ retroviral 19BBz and 1928z CAR plasmids (M.S., Columbia University)^17,39,56^ under a hU6 promoter. CAR constructs coexpressed truncated LNGFR. Single-chain variable fragment sequences against CD22 (EP2912061B1), B7H3 (WO2017044699A1) and L1CAM (US20210085719A1) were cloned into the SFG-19BBz and SFG-1928z vectors using standard molecular biology techniques. Plasmids were used for retrovirus production as previously described^17^. H29 packaging cells were provided by M.S. (Columbia University) and 293Vec-RD114 packaging cells were provided by BioVec Pharma^57^. The PI3Kδ amino acid sequence was obtained from UniProt (O00329) and the respective nucleotide sequence was cloned into the SFG-19BBz or SFG-1928z vector, replacing truncated LNGFR. For ABD screening, T cells were transduced with the retroviral pools to express either 1928z or 19BBz CARs and the sgRNA library. Forty-eight hours after transduction, 2 × 10^6^ T cells were resuspended in P3 primary cell buffer (Lonza) and electroporated with 10 µg of the respective base editor mRNA using Lonza Nucleofector device (EC115 program). Edited CAR T cell pools were subjected to weekly Nalm6 stimulations (effector-to-target ratio (E:T) 2:1) for a total of three stimulations. Genomic DNA was purified using the DNeasy blood and tissue kit (Qiagen) from start-point and end-point samples and edited loci were amplified by PCR using NGS 5′ Illumina specific 30-bp partial adaptors. For *PIK3CD*, exons 1 and 2 spanning the screened p110δ ABD were amplified; for *CD19* and *RASA2*, regions surrounding the respective sgRNA binding sites were targeted. Amplicons were sequenced by AmpliconEZ targeted amplicon deep sequencing (Azenta). Data analysis was carried out using the public Galaxy platform (https://usegalaxy.org/). Adaptor removal and quality trimming were performed using the TrimGalore application with a threshold Phred-score of 25 (https://github.com/FelixKrueger/TrimGalore). Reads were aligned to the reference sequences around the amplified loci using the BWA-MEM2 alignment algorithm^58^ and variants were called using the LoFreq variant caller application^59^. Mutations that were represented under 0.4% at the assay start point were excluded from further analysis. Mutational prevalence was normalized to the CAR T cell rate and analyzed as the fold change (FC) compared to start-point prevalence.

Haplotype analysis was performed by extracting reads harboring specific nucleotide substitutions corresponding to the amino acid changes L32P or E81K. Therefore, we implemented a custom filtering pipeline using pysam library (version 0.21.0; https://github.com/pysam-developers/pysam)^60^. Reads containing the respective codon-altering variants were isolated and subsequently reanalyzed to identify co-occurring mutations within the same sequencing reads.

### Base editing in primary human CAR T cells with synthetic sgRNAs

T cells were isolated and stimulated as described above. Then, 48 h after, 3 × 10^6^ T cells were resuspended in 100 µl of buffer T and electroporated with 10 µg of mRNA and 5 µg of each synthetic sgRNA (Integrated DNA Technologies) resuspended in IDTE buffer (Integrated DNA Technologies, 11-05-01-05) at 1,400 V (three pulses, 10 ms) using the Neon electroporator device. Mock-edited T cells were electroporated with scrambled sgRNA (GCACTACCAGAGCTAACTCA); *TRBC1/2* KO and E81K and L32P substitutions were induced by use of sgRNA sequences CCCACCAGCTCAGCTCCACG (AncBE4max), GCTCTTGCTGCTCCGCTGTC (AncBE4max) and AAGTTCAGGTAGACCCCTGT (ABEmax), respectively. Cells were rested in antibiotic-free X-Vivo15 supplemented with cytokines for 24 h and transduced with retroviral supernatant in RPMI-1640 (Gibco) with 10% FBS supplemented with cytokines by centrifugation on retronectin-coated (Takara, T110A) plates (300*g*, 1 h, 34 °C). CAR T cells with *TRBC1/2* KO were enriched with human CD3 microbeads (Miltenyi Biotec, 130-097-043).

Base-editing efficiency was routinely assessed by Sanger sequencing followed by analysis with EditR^61^ default settings (https://moriaritylab.shinyapps.io/editr_v10/).

Substitutions co-occuring with L32P and E81K were analyzed using AmpliconEZ targeted amplicon deep sequencing (Azenta) as described above.

SUP-T1s were electroporated at 1,400 V (three pulses, 10 ms) using the Neon electroporator device, seeded in a single-cell suspension and sequenced for homozygous E81K substitution.

### Single-cell DNA isolation

First, 7 days after transduction, unstimulated CAR T cells were single-cell-sorted on the basis of LNGFR expression using a MA900 multiapplication cell sorter (Sony). Single-cell DNA was isolated and amplified using REPLI-g single-cell kit according to the manufacturer’s instructions. Subsequently, amplified DNA was used for *PIK3CD* exon 1 PCR amplification followed by Sanger sequencing.

### Antigen stimulation and proliferation assays

CAR T cells (LNGFR^+^) were weekly stimulated with target cells in RPMI-1640 + FBS without cytokines (E:T = 2:1, unless otherwise indicated).

### Mouse systemic tumor model

NOD/SCID/*Il2rγ*-null male and female mice (6–9 weeks old; Charles River, strain code 614, NSG) were used for experiments. All mice were housed in pathogen-free conditions with a housing temperature of 22 ± 1 °C, 55 ± 5% humidity and a 12-h dark–light cycle. All ethical regulations and animal use guidelines were followed. Mouse protocols were approved by the Regierungspräsidium Tübingen and Institutional Animal Care and Use Committee of Zhejiang University (R02/22G, R03/22G, R05/23G and no. 20220178). A total of 0.5 × 10^6^ luciferase–GFP-expressing Nalm6 or Sh-Sy5y cells were administered by tail-vein injection followed by intravenous (i.v.) injection of 3 × 10^6^ 19BBz and 1 × 10^6^ (B7H3-BBz) or 0.5 × 10^6^ (B7H3-28z) CAR T cells 4 days later, respectively. Tumor rechallenge experiments were performed by repetitive i.v. injection of 1 × 10^6^ Nalm6 cells at the indicated time points. Tumor-bearing mice were killed according to signs of morbidity, based on approved scoring sheets involving evaluation of face grimace, posture, weight loss (>20%), respiration, activity, social behavior and grooming. Tumor-free mice that had to be killed for unknown reasons were excluded. For assessment of tumor burden, mice were intraperitoneally administered 150 mg kg^−1^ D-luciferin potassium salt (GoldBio, LUCK-1g) dissolved in sterile PBS for 10 min, followed by imaging under isoflurane anesthesia^62^. Bioluminescence imaging was performed on the IVIS Lumina III system (PerkinElmer) and signal intensity was quantified using Living Image analysis software (version 4.7.4; PerkinElmer) as total flux (photons per s) from consistent regions of interest. To reduce spillover during bioluminescence imaging, we imaged treatment groups together whenever possible.

### Cytotoxicity assays

Ex vivo cytotoxicity was assessed through the generation of single-cell suspensions from spleen, followed by overnight incubation in RPMI-1640 + FBS. In vitro generated or ex vivo obtained CAR^+^ effectors were cocultured with the respective target cells in triplicate at the indicated E:T ratios in 96-well plates with 2 × 10^4^ target cells per well. Maximal luciferase expression was determined using only target cells; Triton X-100-treated cells were used to assess background levels. After 18 h of incubation, 100 μl of luciferase substrate (D-luciferin; Goldbio) was directly added to each well. Emission was detected and lysis was calculated as (1 − Em_sample_/Em_max_) × 100.

### 3D migration cytotoxicity assay and time-lapse microscopy assay

Anti-L1CAM-BBz CAR T cells were used after twice-weekly stimulations with Sh-Sy5y (E:T = 2:1). H2B–mGFP-expressing Sh-Sy5y cells were subconfluently seeded in a black 96-well imaging plate and were overlaid with a 3D collagen gel (PureCol, concentration: 1.7 mg ml^−1^) containing the respective preactivated CAR T cells stained with CellTracker Red CMTPX dye (Thermo Fisher, C34552). After polymerization, T cell dynamics were recorded by time-lapse bright-field microscopy (frame rate = 55 s) for 42–45 h at 37 °C and 5% CO_2_. The 2 × 2 mosaic images were obtained with an epifluorescence microscope (LeicaMicrosystems, Thunder 3D Assay) with hardware-based auto focus control using a Leica ×20 (numerical aperture: 0.95) air objective and computationally merged using the Leica LasX software. Whole-well end-point images were acquired after 4 days using a Tecan SparkCyto. Images were split and segmented by StarDist (https://link.springer.com/chapter/10.1007/978-3-030-00934-2_30, https://ieeexplore.ieee.org/document/9093435); apoptotic cells were excluded and living cells were counted using FIJI. E:T cell interactions were quantified by automatic analysis based on vicinity. For speed quantification, T cells that could be followed for >30 min were included in the statistical analysis. Cells were segmented and tracked using TrackMate FIJI plug-in^63^ with Stardist segmentation and LAP Tracker tracking^64^. T cell analysis was performed using R Studio based on celltrackR^65^ and the work package (https://github.com/juliaquach02/cellcontacts) to detect E:T cell contacts. An interaction or contact was defined as three frames (155 s) and a maximum distance of 7 µm between T cell and tumor cell. Apoptotic events and T cell contacts until tumor cell apoptosis were quantified by manual analysis. Data were evaluated on the original image set.

### Flow cytometric analysis

Cell-surface staining of single-cell suspensions was performed in fluorescence-activated cell sorting (FACS) buffer (PBS with 2% FBS and 2 mM EDTA) for 20 min at room temperature.

Mitochondrial mass was determined by incubation with 10 nM MitoTracker orange CMTMRos (Invitrogen) in culture medium for 30 min at 37 °C, followed by cell-surface staining.

For assessment of intracellular protein levels, effector and target cells were cocultured (E:T = 1:1, 4 h, 37 °C) in the presence of 3 µg ml^−1^ brefeldin A and 2 µM monensin (both Tonbobio), followed by viability and surface staining in PBS for 25 min. Cells were fixed with IC fixation buffer (eBioscience) for 20 min at room temperature. Intracellular staining was performed in 1× permeabilization buffer (eBioscience) for 30 min at 4 °C.

For glucose uptake experiments, cells were analyzed after final stimulation with Nalm6 cells (E:T = 2:1). Cells were washed and resuspended in glucose-free RPMI-1640 + FBS supplemented with 100 µg ml^−1^ 2-NBD-glucose (Abcam, ab146200) to a cell concentration of 3 × 10^6^ cells per ml. Cells were incubated for 10 min at 37 °C, washed with ice-cold PBS and stained extracellularly.

For phosphoflow analysis, cells were seeded in cytokine-free RPMI-1640 for 2 h and subsequently stimulated with Nalm6 cells for at an (E:T = 1:1). After fixation with 100 µl of warm 4% paraformaldehyde for 15 min, cells were washed and permeabilized in 300 µl of 90% ice-cold methanol for 10 min at 4 °C and washed twice. Subsequently, cells were incubated with phosphoantibodies for 1 h at room temperature, washed and stained with secondary antibody for 30 min at room temperature.

For measurement of antigen surface levels, Quantum Simply Cellular antimouse IgG (Bangs Laboratories) was performed and calculated according to the manufacturer’s instructions.

For CAR T cell sorts, unstimulated CAR T cells were sorted on a MA900 multiapplication cell sorter (Sony, 100-µm chip). LNGFR^+^ CAR T cells were sorted for indicated CAR T cell subsets (naive, CD45RA^+^CD62L^+^; central memory, CD45RA^−^CD62L^+^; effector memory, CD45RA^−^CD62L^−^; effector, CD45RA^+^CD62L^−^).

**Extended Data Fig. 10:**
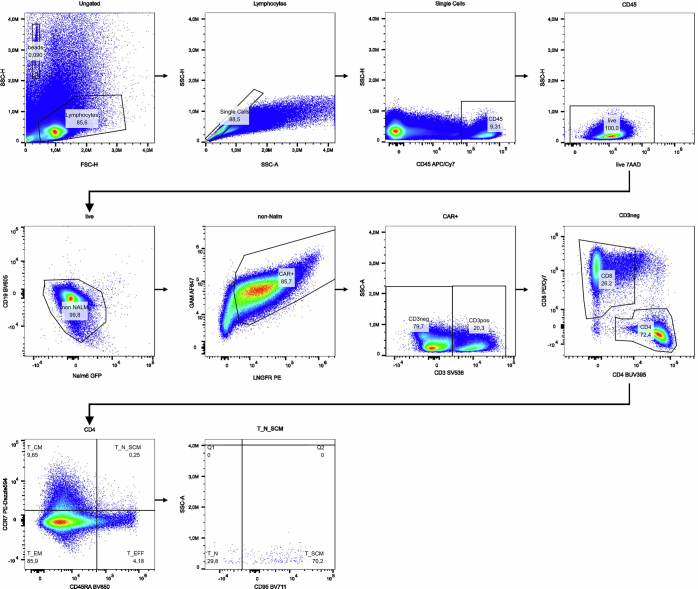
Exemplary gating strategy for in vivo samples. Exemplary gating strategy for CAR T cell differentiation of E81K-edited 19BBz CAR T cells from bone marrow samples 16 days after T cell infusion into Nalm6-bearing mice.

Samples obtained from in vivo experiments were blocked using mouse FcR Blocking Reagent (Miltenyi Biotec, 130-092-575) in addition to the CAR and surface staining. Blood samples were collected in EDTA-coated microtainer and washed with cold FACS buffer. FACS lysis solution (BD, 349202) was added to lyse red blood cells. CountBright absolute counting beads (Invitrogen) were added to the samples before acquisition. The gating strategy for in vivo samples is exemplarily shown in Extended Data Fig. 10. In all experiments involving *TRBC1/2* KO, the CAR^+^ cells for subsequent analysis were determined as the proportion of CD3^−^ T cells.

Flow cytometry and western blot antibodies and reagents are reported as Supplementary Tables 11 and 12. All samples were acquired on a five-laser Cytek Aurora spectral cytometer with automated compensation calculation. Fluorescence − 1 controls were acquired for accurate gating. Data analysis was performed using FlowJo (version 10.8.0).

### Western blot

For immunoblotting, SUP-T1 or T cells were seeded at 2 × 10^6^ cells per ml in fresh RPMI-1640 + FBS. Cells were either left untreated or incubated with Nalm6 cells (E:T = 2:1). Cells were washed with ice-cold PBS and spun down (300*g*, 5 min, 4 °C). Pellets were resuspended in supplemented RIPA buffer (protease inhibitor (Roche), NaF, Na_4_P_2_O_7_ and Na_3_VO_4_) and incubated for 15 min on ice with repetitive vigorous mixing. Protein concentration was measured using BioRad protein assay. Then, 40–60 µg of protein lysate was loaded onto 10% Tris–glycine SDS Gels followed by transfer to nitrocellulose membrane. Membranes were blocked in 5% BSA or milk in TBST; thereafter, primary antibodies were incubated at 4 °C overnight and secondary antibodies were incubated for 2 h at room temperature.

### scRNA-seq library preparation and sequencing

First, 19BBz CAR T cells carrying either a *TRBC1/2* stop mutation and/or the E81K base edit were stimulated twice with Nalm6 cells (E:T = 2:1). Then, 48 h after the last stimulation, living CAR T cells were single-cell-sorted (LNGFR^+^CD3^−^) and single-cell sequenced (CeGaT). Cell count and viability (>90%) were determined using a Cellaca MX cell counter (Revvity). The scRNA-seq library was prepared using Chromium NextGEM single-cell 3′ kit, version 3.1 (10x Genomics) according to the manufacturer’s instructions. Briefly, the cell suspension was loaded into a Chromium NextGEM ChipG aiming for a cell recovery of 10,000 cells per sample. For cDNA amplification, 11 PCR cycles were used and the final sample index PCR was performed with 12 cycles. Libraries were sequenced using a NovaSeq X Plus 1.5B flow cell (mean reads per cell: 19BBz TRBC, 113,977; 19BBz E81K, 121,606).

### scRNA-seq analysis

Demultiplexing of the sequencing reads was performed with bcl2fastq (version 2.20). For each sample, FASTQ files generated with bcl2fastq were subsequently processed with the Cell Ranger software (version 7.1.0) provided by 10x Genomics.

Processing and analysis of scRNA-seq data were performed with Scanpy (version 1.11.1)^66^. Genes with <20 total counts and cells with >100,000 total or 10% mitochondrial gene counts, <2,000 distinct genes or 10% ribosomal gene counts were filtered out. The gene expression matrix was normalized to the median of total counts per cell, log-transformed and scaled per gene. The 2,000 most highly variable genes were determined with the ‘seurat_v3’ method on the basis of raw counts^67^. Scaled expression of these highly variable genes was used to compute a principal component analysis and a neighborhood graph with 30 nearest neighbors was constructed on the basis of 50 principal components using cosine similarity. Leiden clustering (resolution 1.0) was performed and uniform manifold approximation and projection (UMAP) embedding was computed with default parameters for visualization.

For cell type annotation, differentially expressed genes (DEGs) between Leiden clusters were determined using a Wilcoxon rank-sum test on log-normalized counts with the Scanpy function ‘rank_genes_groups’ (ref. ^66^). Overrepresentation analysis of differentially upregulated genes for each cluster (false-discovery-rate-corrected *P* value < 0.05, log_2_FC > 0.5) and GSEA^68^ based on differential expression scores were performed using GSEApy (version 1.1.8)^69^ with the Kyoto Encyclopedia of Genes and Genomes pathway, Gene Ontology biological process, MSigDB Hallmark, MSigDB C7 immunologic signature and additional exhaustion-related gene sets^45^ to aid with functional annotations. Clusters with similar marker gene expression profiles (Extended Data Fig. 7c) were aggregated into functional cell types. Gene module expression^46^ was scored using the Scanpy function ‘score_genes’ (ref. ^66^) on the basis of log-normalized counts.

### Untargeted metabolomics and targeted metabolite analysis

First, 19BBz CAR T cells with a *TRBC1/2* stop mutation and/or E81K substitution were twice stimulated with Nalm6 cells (E:T = 2:1). Then, 24 h after last stimulation, the absence of Nalm6 cells was controlled by flow analysis and 1 × 10^−7^ to 1.2 × 10^−7^ CAR T cells were thoroughly washed followed by quenching with 400 µl of ice-cold methanol (≥99.9%) and storage at −80 °C. Metabolite extraction was performed on ice using a two-phase extraction with methanol, methyl *tert*-butyl ether and H_2_O as previously described^70^. Liquid chromatography (LC)–mass spectrometry (MS) analysis was performed using a chromatography system coupled to a timsTOF Pro2 mass spectrometer (Bruker Daltonics) equipped with a vacuum-insulated pressure-heated electrospray ionization source (Bruker Daltonics). Hydrophilic interaction LC (HILIC) was performed using a BEH amide column (150 × 2.1 mm, 1.7 µm; Waters). To manually assess signal linearity, a pooled quality control sample was injected (0.25–5 µl). Mass and ion mobility were recalibrated using sodium formate and Agilent’s ESIS tuning solution per run. The MS analysis for nontargeted metabolomics was performed using parallel accumulation serial fragmentation mode with data-dependent MS/MS acquisition and trapped ion mobility spectrometry stepping, following the four-dimensional (4D) metabolomics standard method in TimsControl software (Bruker Daltonics). HILIC was run in both ionization modes; reverse phase LC was run in negative mode only.

Raw data were processed in MetaboScape 2025b using the T-ReX 4D algorithm and matched to commercial and in-house target lists and libraries (Bruker HMDB 2.0, METLIN-CCS, PNNL, IROA MSMLS). Metabolite identification followed Metabolomics Standards Initiative level 2 criteria (*m*/*z* deviation < 2.0 ppm, mSigma < 20, MS/MS score < 900 or collision cross-section deviation < 1%). Features had to be ≥3× blank, detected in ≥5 samples and have ≥1,500 ion counts and ≥125 4D points. Recursive extraction and quality-control-based batch correction were applied.

Data preprocessing and further statistical processing were performed using MetaboAnalyst 6.0. Missing values were imputed as one fifth of the minimum positive value, normalized by probabilistic quotient normalization, log_10_-transformed and merged across HILIC and RP modes. Redundant features were retained from the method with fewer missing values and higher intensity. Samples were run in five technical replicates unless stated otherwise. Hierarchical clustering was performed using Euclidean distance and the ward.D2 linkage method. Joint transcriptomics from overall scRNA analysis and metabolomic pathway analysis was conducted using the MetaboAnalyst 6.0. For this analysis, 222 upregulated genes (adjusted *P* value < 0.05, FC > 1) and 27 upregulated metabolites (*P* value < 0.05 in at least one donor) were included. Pathway enrichment was assessed using hypergeometric testing and pathway topology was evaluated on the basis of betweenness centrality.

Absolute NADH levels of CAR T cells were measured 24 h after second antigen stimulation using a NAD^+^/NADH assay kit (Sigma-Aldrich, MAK460) according to the manufacturer’s instructions.

### Seahorse analysis

Metabolic profiling was measured using a flux analyzer (Seahorse XF Pro, Agilent) according to the manufacturer’s instructions. A total of 2 × 10^5^ T cells per well were seeded in 5–8 replicates in 180 µl of Seahorse XF RPMI medium (Agilent) containing 2 mM L-glutamine, 1 mM sodium pyruvate and 10 mM D-glucose in poly(D-lysine)-coated 96-well microplates. After 30 min of incubation at 37 °C, mitochondrial respiration analysis was performed using the Seahorse XF cell mito stress test kit (Agilent, 103015-100) according to the manufacturer’s recommendation. Basal oxygen consumption was calculated from the difference in oxygen consumption rate (OCR) before oligomycin treatment and after rotenone + antimycin A treatment. Maximal oxygen consumption was calculated from the difference of the OCR after rotenone + antimycin A treatment and after FCCP treatment. Basal and maximal extracellular acidification rate (ECAR) was defined as the ECAR measured before and after oligomycin addition. Spare respiratory capacity and spare ECAR were defined as the difference between maximal and basal oxygen consumption and ECAR, respectively.

### Multiplex immunoassay and cytokine secretion analysis

For cytokine quantification, the LEGENDplex human essential immune response panel (BioLegend) was performed according to the manufacturer′s instructions. Data analysis was performed according to manufacturer’s instructions using the LEGENDplex data analysis software. In brief, serum samples from mice were collected on day 16 and used both undiluted and in a 1:100 dilution for the assay. For in vitro experiments, CAR T cells and target cells were cocultured (24 h, E:T = 1:1). All measurements were performed in duplicates.

### RNA extraction and bulk RNA-seq analysis

CAR T cells were harvested either unstimulated or after twice-weekly stimulations with Nalm6. Then, 4 days after the last stimulation, no Nalm6 cells were detectable in the culture as determined by flow cytometry. All cells were washed twice with ice-cold PBS and RNA was directly extracted using the RNeasy mini kit with RNase-Free DNase set (Qiagen) according to the manufacturer’s instructions.

### Transcriptomic data analysis of bulk RNA-seq

Quality control, read mapping and counting were performed using the Nextflow-based nf-core/rnaseq (version 3.12.0) pipeline (https://nf-co.re/rnaseq). Quality control assessment of the raw data was performed using FastQC (version 0.11.9) and RSeQC (version 3.0.1). Reads were mapped to the reference genome (GRCh38) using the STAR aligner (version 2.6.1d)^71^. Quantification of raw gene expression was performed with Salmon (version 1.10.1)^72^ and downstream analysis was performed with the Nextflow-based rnadeseq (version 2.2.0) pipeline (https://github.com/qbic-pipelines/rnadeseq). This workflow integrates DESeq2 (version 1.40.2)^73^ for differential expression analysis used in the R language (version 4.3.1). Genes with an adjusted *P* value ≤ 0.05 were considered differentially expressed. The DEG lists were obtained from simple pairwise comparisons extracted from a linear model. The DEG lists, together with their associated log_2_FC were fed into gprofiler2 for pathway enrichment analysis. The gene selection for the comparative analysis between 19BBz CAR T cells with and without the E81K substitution in Extended Data Fig. 6h was based on the FOXO3-dependent gene set reported by Litvak et al.^41^ (Fig. 1a). Specifically, the top 30 genes upregulated in *FOXO3*-KO versus WT conditions and expressed across both human and murine species were selected for further analysis. For the GSEA, fgsea (version 1.28.0)^71^ was used to run the analysis on the output of DESeq2. The reference gene list used included two custom gene sets and MSigDB (version 7.2) Hallmark gene sets.

### Histological analysis

All tissues were fixed in 4% buffered formalin and embedded in paraffin. For histology 3–5 µm-thick sections were cut and stained with hematoxylin and eosin. The histologic samples were analyzed by an experienced pathologist (L.Q.-M.). All samples were scanned with the Ventana DP200 (Roche) and processed with the Image Viewer MFC Application. Final image preparation was performed with Adobe Photoshop 2024.

### Statistical analysis

Any additional statistical analyses not detailed above were conducted using Prism 7 (GraphPad). Mouse condition and survival were observed by an operator who was blinded to treatment groups in addition to the main investigator who was not blind to group allocation. Tumor burden was measured by a blinded operator; analysis of data was not performed in blinded fashion. No data were excluded throughout the studies. No blinding was performed for in vitro experiments. No statistical sample size was predetermined. Mice were randomly allocated to the respective groups after ensuring similar tumor burden. Comparisons between two groups were conducted using two-sided Student’s *t*-tests. When normality could not be assumed (Shapiro–Wilk test), the Wilcoxon matched-pairs signed-rank test was applied for paired data. For values normalized to their respective control, two-sided one-sample *t*-tests were used if the data were normally distributed; otherwise, a two-sided one-sample Wilcoxon test was applied. For comparing multiple groups, we applied one-way analysis of variance. For in vivo experiments, overall survival was analyzed using Kaplan–Meier curves and survival differences between groups were assessed with the log-rank (Mantel–Cox) test. *P* values < 0.05 were considered statistically significant. Specific statistical tests used are detailed in the corresponding figure legends. Scientific illustrations were generated by J. Zenker with BioRender.com drafts.

### Reporting summary

Further information on research design is available in the Nature Portfolio Reporting Summary linked to this article.

## Supplementary information

Reporting Summary

Supplementary Tables 1–12 Overview sheet. Supplementary Tables 1–8: DEGs of all annotated cluster of the scRNA-seq analysis with E81K-modified and nonmodified 19BBz CAR T cells after two antigen stimulations. Supplementary Tables 9 and 10: Sequences of used sgRNAs for ABE and CBE screening. Supplementary Tables 11 and 12: Used antibodies and other reagents.

## Source data

Source Data Source data for all figures and Extended Data figures.

Source Data Extended Data Fig. 2 Unprocessed western blots.

## Data Availability

All data generated and supporting the findings are available within the article or its Supplementary Information. Structural data related to Fig. 1 are available from the Protein Data Bank under accession code PDB 7JIS. The raw data from the metabolomics can be accessed through Zenodo (10.5281/zenodo.17426175)^74^. The processed single-cell and bulk RNA-seq data were deposited to the ArrayExpress repository under the accession numbers E-MTAB-15746 and E-MTAB-15749. The raw data obtained from bulkRNAseq and scRNA-seq analysis were deposited to Zenodo (10.5281/zenodo.17293317 (ref. ^75^) and 10.5281/zenodo.17292714 (ref. ^76^)) and can be provided upon reasonable request to the corresponding authors. Access of raw data is restricted in accordance with the ethical approval and participant consent requirements. Further information and materials will be made available upon reasonable request. Source data are provided with this paper.

## Code availability

All codes used for analysis are referenced in the method section and are publicly available. The newly developed code used for live-cell imaging analysis is publicly available (https://github.com/juliaquach02/cellcontacts).
