# Supplementary material for: CAR-adapted PIK3CD base editing enhances T cell anti-tumor potency
Source: Nat Cancer. 2026 Jan 6;7(2):368–83. doi: 10.1038/s43018-025-01099-7 (PMC12948676; doi:10.1038/s43018-025-01099-7)
Supplement: Supplementary file 1 — Reporting Summary [file 43018_2025_1099_MOESM1_ESM.pdf]

nature portfolio

Corresponding author(s): *Josef Leibold, Judith Feucht*

Last updated by author(s): *Nov 6, 2025*

Reporting Summary

Nature Portfolio wishes to improve the reproducibility of the work that we publish. This form provides structure for consistency and transparency in reporting. For further information on Nature Portfolio policies, see our Editorial Policies and the Editorial Policy Checklist.

Statistics

For all statistical analyses, confirm that the following items are present in the figure legend, table legend, main text, or Methods section.

n/a	Confirmed	
☐	☒	The exact sample size (*n*) for each experimental group/condition, given as a discrete number and unit of measurement
☐	☒	A statement on whether measurements were taken from distinct samples or whether the same sample was measured repeatedly
☐	☒	The statistical test(s) used AND whether they are one- or two-sided<br>*Only common tests should be described solely by name; describe more complex techniques in the Methods section.*
☒	☐	A description of all covariates tested
☐	☒	A description of any assumptions or corrections, such as tests of normality and adjustment for multiple comparisons
☐	☒	A full description of the statistical parameters including central tendency (e.g. means) or other basic estimates (e.g. regression coefficient) AND variation (e.g. standard deviation) or associated estimates of uncertainty (e.g. confidence intervals)
☐	☒	For null hypothesis testing, the test statistic (e.g. *F*, *t*, *r*) with confidence intervals, effect sizes, degrees of freedom and *P* value noted<br>*Give P values as exact values whenever suitable.*
☒	☐	For Bayesian analysis, information on the choice of priors and Markov chain Monte Carlo settings
☒	☐	For hierarchical and complex designs, identification of the appropriate level for tests and full reporting of outcomes
☒	☐	Estimates of effect sizes (e.g. Cohen's *d*, Pearson's *r*), indicating how they were calculated

Our web collection on statistics for biologists contains articles on many of the points above.

Software and code

Policy information about availability of computer code

Data collection	*Cytek Aurora spectral cytometer, PerkinElmer IVIS Imaging System, Microsoft Excel 2019, BioRad ChemiDoc XRS+, Aglient Seahorse XF Pro Analyzer, LeicaMicrosystems Thunder 3D Assay, Tecan Spark Cyto, Ventana DP200, Bruker HyStar; TimsControl; NovaSeq X Plus 1.5b, NovaSeq 6000*
Data analysis	*GraphPad Prism 7, Microsoft Excel 2019, FlowJo v10.8.0, ImageJ Fiji, BioRad ImageLab 6.1, R package fgsea v1.28.0, DESeq2 v1.40.2, rnadeseq v2.2.0, Salmon v1.10.1, FastQC v0.11.9, RSeQC v3.0.1, STAR aligner v2.6.1d, Image Viewer MFC Application, Adobe Photoshop 2024; MetaboScape 2024b; MetaboAnalyst web server (Version 6.0); bcl2fastq v2.20; Cell Ranger software v7.1.0; Scanpy; GESApy, newly developed code used for live cell imaging analysis was made publicly available (https://github.com/juliaquach02/cellcontacts)*

For manuscripts utilizing custom algorithms or software that are central to the research but not yet described in published literature, software must be made available to editors and reviewers. We strongly encourage code deposition in a community repository (e.g. GitHub). See the Nature Portfolio guidelines for submitting code & software for further information.

Data

Policy information about availability of data

All manuscripts must include a data availability statement. This statement should provide the following information, where applicable:

- Accession codes, unique identifiers, or web links for publicly available datasets
- A description of any restrictions on data availability
- For clinical datasets or third party data, please ensure that the statement adheres to our policy

The processed single-cell and bulk RNA sequencing data have been deposited in the ArrayExpress repository under the accession numbers E-MTAB-15746 (https://www.ebi.ac.uk/biostudies/ArrayExpress/studies/E-MTAB-15746) and E-MTAB-15749 (https://www.ebi.ac.uk/biostudies/ArrayExpress/studies/E-MTAB-15749). The raw data obtained form bulkRNAseq and scRNAseq analysis have been deposited on Zenodo (https://zenodo.org/records/17293318, https://zenodo.org/records/17292715) and will be provided upon reasonable request to the corresponding authors. Access of raw data is restricted in accordance with the ethical approval and participant consent requirements.
Metabolomics raw data is available at Zenodo: https://zenodo.org/records/17426176. Further information and materials will be made available upon reasonable request.

Research involving human participants, their data, or biological material

Policy information about studies with human participants or human data. See also policy information about sex, gender (identity/presentation), and sexual orientation and race, ethnicity and racism.

Reporting on sex and gender	*Buffy coats were obtained from unidentified donors of both sex. Blood samples of pre-treated tumor patients wer taken of both sex.*
Reporting on race, ethnicity, or other socially relevant groupings	*N/A*
Population characteristics	*All buffy coats and blood samples were obtained from healthy donors older than 18 years.*
Recruitment	*Donors were voluntary blood donors, tumor patients were asked for blood samples according to ethical guidelines by medical personal not involved to this research. None of the authors were involved in selection of human research participants.*
Ethics oversight	*All ethic regulations were followed, ethical approval: 783/2023BO2 and 846/2020BO2 (Ethic commission University of Tuebingen).*

Note that full information on the approval of the study protocol must also be provided in the manuscript.

Field-specific reporting

Please select the one below that is the best fit for your research. If you are not sure, read the appropriate sections before making your selection.

☒ Life sciences ☐ Behavioural & social sciences ☐ Ecological, evolutionary & environmental sciences

For a reference copy of the document with all sections, see nature.com/documents/nr-reporting-summary-flat.pdf

Life sciences study design

All studies must disclose on these points even when the disclosure is negative.

Sample size	*No statistical methods were used to pre-determine sample size. Sample sizes were estimated based on preliminary experiments, with an effort to achieve a minimum of n=4 mice per treatment group. To enhance robustness, we tried to repoduce experiments with at least two donors if possible.*
Data exclusions	*No data were excluded throughout the studies.*
Replication	*All attempts at replication were successful. Experiments were at least reproduced three times besides the live cell imaging which was reproduced once. For in vivo experiments the numbers of tested donors are stated in the respective Figure legend.*
Randomization	*Tumor burden was determined by bioluminescence imaging one day prior to CAR T cell transfer. Since tumor burdens are very even with the NALM6 cell line, no mice were excluded prior to treatment and mice were randomly assigned into treatment groups. Buffy coats were obtained from anonymous healthy donors.*
Blinding	*Mouse condition and survival were observed by an operator who was blinded to treatment groups in addition to the main investigator who was not blind to group allocation. Tumor burden was measured by a blinded operator, analysis of data was not performed in blinded fashion. All data analyses are based on objectively measurable data (fluorescence intensity, tumor burden, cell count, gene expression level). In vitro experiments were not blinded.*

Behavioural & social sciences study design

All studies must disclose on these points even when the disclosure is negative.

Study description	*Briefly describe the study type including whether data are quantitative, qualitative, or mixed-methods (e.g. qualitative cross-sectional, quantitative experimental, mixed-methods case study).*
Research sample	*State the research sample (e.g. Harvard university undergraduates, villagers in rural India) and provide relevant demographic information (e.g. age, sex) and indicate whether the sample is representative. Provide a rationale for the study sample chosen. For studies involving existing datasets, please describe the dataset and source.*
Sampling strategy	*Describe the sampling procedure (e.g. random, snowball, stratified, convenience). Describe the statistical methods that were used to predetermine sample size OR if no sample-size calculation was performed, describe how sample sizes were chosen and provide a rationale for why these sample sizes are sufficient. For qualitative data, please indicate whether data saturation was considered, and what criteria were used to decide that no further sampling was needed.*
Data collection	*Provide details about the data collection procedure, including the instruments or devices used to record the data (e.g. pen and paper, computer, eye tracker, video or audio equipment) whether anyone was present besides the participant(s) and the researcher, and whether the researcher was blind to experimental condition and/or the study hypothesis during data collection.*
Timing	*Indicate the start and stop dates of data collection. If there is a gap between collection periods, state the dates for each sample cohort.*
Data exclusions	*If no data were excluded from the analyses, state so OR if data were excluded, provide the exact number of exclusions and the rationale behind them, indicating whether exclusion criteria were pre-established.*
Non-participation	*State how many participants dropped out/declined participation and the reason(s) given OR provide response rate OR state that no participants dropped out/declined participation.*
Randomization	*If participants were not allocated into experimental groups, state so OR describe how participants were allocated to groups, and if allocation was not random, describe how covariates were controlled.*

Ecological, evolutionary & environmental sciences study design

All studies must disclose on these points even when the disclosure is negative.

Study description	*Briefly describe the study. For quantitative data include treatment factors and interactions, design structure (e.g. factorial, nested, hierarchical), nature and number of experimental units and replicates.*
Research sample	*Describe the research sample (e.g. a group of tagged Passer domesticus, all Stenocereus thurberi within Organ Pipe Cactus National Monument), and provide a rationale for the sample choice. When relevant, describe the organism taxa, source, sex, age range and any manipulations. State what population the sample is meant to represent when applicable. For studies involving existing datasets, describe the data and its source.*
Sampling strategy	*Note the sampling procedure. Describe the statistical methods that were used to predetermine sample size OR if no sample-size calculation was performed, describe how sample sizes were chosen and provide a rationale for why these sample sizes are sufficient.*
Data collection	*Describe the data collection procedure, including who recorded the data and how.*
Timing and spatial scale	*Indicate the start and stop dates of data collection, noting the frequency and periodicity of sampling and providing a rationale for these choices. If there is a gap between collection periods, state the dates for each sample cohort. Specify the spatial scale from which the data are taken*
Data exclusions	*If no data were excluded from the analyses, state so OR if data were excluded, describe the exclusions and the rationale behind them, indicating whether exclusion criteria were pre-established.*
Reproducibility	*Describe the measures taken to verify the reproducibility of experimental findings. For each experiment, note whether any attempts to repeat the experiment failed OR state that all attempts to repeat the experiment were successful.*
Randomization	*Describe how samples/organisms/participants were allocated into groups. If allocation was not random, describe how covariates were controlled. If this is not relevant to your study, explain why.*
Blinding	*Describe the extent of blinding used during data acquisition and analysis. If blinding was not possible, describe why OR explain why blinding was not relevant to your study.*

Did the study involve field work? ☐ Yes ☐ No

Field work, collection and transport

Field conditions	*Describe the study conditions for field work, providing relevant parameters (e.g. temperature, rainfall).*
Location	*State the location of the sampling or experiment, providing relevant parameters (e.g. latitude and longitude, elevation, water depth).*
Access & import/export	*Describe the efforts you have made to access habitats and to collect and import/export your samples in a responsible manner and in compliance with local, national and international laws, noting any permits that were obtained (give the name of the issuing authority, the date of issue, and any identifying information).*
Disturbance	*Describe any disturbance caused by the study and how it was minimized.*

Reporting for specific materials, systems and methods

We require information from authors about some types of materials, experimental systems and methods used in many studies. Here, indicate whether each material, system or method listed is relevant to your study. If you are not sure if a list item applies to your research, read the appropriate section before selecting a response.

Materials & experimental systems

n/a	Involved in the study	
☐	☒	Antibodies
☐	☒	Eukaryotic cell lines
☒	☐	Palaeontology and archaeology
☐	☒	Animals and other organisms
☒	☐	Clinical data
☒	☐	Dual use research of concern
☒	☐	Plants

Methods

n/a	Involved in the study	
☒	☐	ChIP-seq
☐	☒	Flow cytometry
☒	☐	MRI-based neuroimaging

Antibodies

Antibodies used

Flow cytometry antibodies used in this study are specific for
anti-human CD4 Brilliant Ultra Violet 395 (563550) BD (1:200)
anti-human CD19 Brilliant Violet 605 (562653) BD (1:200)
anti-human CD45 APC/Cy7 (557833) BD (1:100)
anti-human CD279 (PD-1) Brilliant Violet 480 (566112) BD (1:100)
anti-human IFN-γ Brilliant Ultra Violet 737 (612845) BD (1:100)
anti-human CD271 (LNGFR) Alexa Fluor 647 (560326) BD (1:150)
anti-human CD271 (LNGFR) PE (557196) BD (1:75)
anti-human CD3 SparkViolet538 (300484) BioLegend (1:200)
anti-human CD3 Brilliant Violet 605 (344835) BioLegend (1:200)
anti-human CD3 APC-Cy7 (344818) BioLegend (1:200)
anti-human CD19 Brilliant Violet 421 (302234) BioLegend (1:100)
anti-human CD25 APC/Fire 810 (356150) BioLegend (1:100)
anti-human CD69 Alexa Fluor 647 (310918) BioLegend (1:100)
anti-human CD62L Brilliant Violet 421 (304828) Biolegend (1:100)
anti-human CCR7 PE/Dazzle594 (353236) BioLegend (1:100)
anti-human CD45RA Brilliant Violet 650 (304136) BioLegend (1:100)
anti-human CD57 PE/Dazzle594 (359619) BioLegend (1:100)
anti-human CD39 PerCP/Cy5.5 (328218) BioLegend (1:100)
anti-human CD95 (Fas) Brilliant Violet 711 (305644) BioLegend (1:100)
anti-human CD366 (Tim-3) Brilliant Violet 785 (345032) BioLegend (1:100)
anti-human CD223 (LAG-3) Brilliant Violet 650 (369316) BioLegend (1:100)
anti-human TIGIT KIRAVIA Blue 520 (372732) BioLegend (1:100)
anti-human IL-2 PerCP (500350) BioLegend (1:100)
anti-human TNF-α APC/Cy7 (502944) BioLegend (1:100)
anti-human Granzyme B Pacific Blue (372218) BioLegend (1:100)
anti-human CD183 (CXCR3) Alexa Fluor 700 (353742) BioLegend (1:100)
anti-human CD11a/CD18 (LFA1) Brilliant Violet 421 (363408) BioLegend (1:100)
anti-human CD171 (L1CAM) PE (371604) BioLegend (1:100)
anti-human KLRG1 APC/Cy7 (367724) BioLegend (1:100)
anti-human CD8a PE/Cy7 (25-0087-42) Invitrogen (1:200)
anti-human CD22 PE (302506) BioLegend (1:100)
anti-human B7H3 PE/Dazzle 594 (351012) BioLegend (1:200)
p-Akt (S473) (4056S) Cell Signalling technolgies (1:200)
p-Akt (T308) (13038S) Cell Signalling technolgies (1:1000)

p-S6 ribosomal protein (S235/236) (4858S) Cell Signalling technolgies (1:200)
p-ERK1/2 (MA5-15174) Invitrogen (1:1000)
anti-mouse AffiniPure™ F(ab')2 Fragment Goat Anti-Mouse IgG, F(ab')2 fragment specific Alexa Fluor 647 (115-606-072) JacksonImmunoResearch (1:1000)
anti-mouse AffiniPure™ F(ab')2 Fragment Goat Anti-Mouse IgG, F(ab')2 fragment specific Alexa Fluor 594 (115-586-072) JacksonImmunoResearch (1:1000)
7-AAD BD (51-68981E) 1:200
For Western Blot following antibodies were used:
Tubulin (T9026) Sigma-Aldrich (life sciences) (1:2000)
p-Akt (S473) (4056S) Cell Signalling technolgies (1:1000)
p-Akt (T308) (13038S) Cell Signalling technolgies (1:1000)
p-S6 ribosomal protein (S235/236) (4858S) Cell Signalling technolgies (1:1000)
p-ERK1/2 (MA5-15174) Invitrogen (1:1000)
anti-mouse Peroxidaseconjugated AffiniPure Goat Anti-Mouse IgG (H+L) (115-035-062) Jackson Immuno Research (1:10000)
anti-rabbit Peroxidase-conjugated AffiniPure Goat Anti-Rabbit IgG (H+L) (111-035-0450) Jackson Immuno Research (1:10000)

Validation: All the antibodies are validated for use in flow cytometry and for immunoblot analysis. Data are available on the manufacturer's website.

Eukaryotic cell lines

Policy information about cell lines and Sex and Gender in Research

Cell line source(s)	ATCC, partner laboratories
Authentication	Sh-SY5Y cell line was authenticated by ShortTandemRepeat (STR) analysis by MicroSynth.
Mycoplasma contamination	All cell lines were routinely PCR-tested for mycoplasma and were found to be negative.
Commonly misidentified lines (See ICLAC register)	No commonly misidentified cell lines were used.

Palaeontology and Archaeology

Specimen provenance	Provide provenance information for specimens and describe permits that were obtained for the work (including the name of the issuing authority, the date of issue, and any identifying information). Permits should encompass collection and, where applicable, export.
Specimen deposition	Indicate where the specimens have been deposited to permit free access by other researchers.
Dating methods	If new dates are provided, describe how they were obtained (e.g. collection, storage, sample pretreatment and measurement), where they were obtained (i.e. lab name), the calibration program and the protocol for quality assurance OR state that no new dates are provided.

☐ Tick this box to confirm that the raw and calibrated dates are available in the paper or in Supplementary Information.

Ethics oversight	Identify the organization(s) that approved or provided guidance on the study protocol, OR state that no ethical approval or guidance was required and explain why not.

Note that full information on the approval of the study protocol must also be provided in the manuscript.

Animals and other research organisms

Policy information about studies involving animals; ARRIVE guidelines recommended for reporting animal research, and Sex and Gender in Research

Laboratory animals	NSG (NOD.Cg-Prkdc<scid>Il2rg<tm1Wjl>SzJ) mice were male and female, 6-9 weeks old and obtained from Charles River
Wild animals	N/A
Reporting on sex	N/A
Field-collected samples	N/A
Ethics oversight	Animal studies were performed in accordance with approved ethical requirements, approvals R02/22G, R03/22G, R05/23G (Regierungspräsidium Tübingen) and No.20220178 (Institutional Animal Care and Use Committee of Zhejiang University).

Note that full information on the approval of the study protocol must also be provided in the manuscript.

Clinical data

Policy information about clinical studies

All manuscripts should comply with the ICMJE guidelines for publication of clinical research and a completed CONSORT checklist must be included with all submissions.

Clinical trial registration	*Provide the trial registration number from ClinicalTrials.gov or an equivalent agency.*
Study protocol	*Note where the full trial protocol can be accessed OR if not available, explain why.*
Data collection	*Describe the settings and locales of data collection, noting the time periods of recruitment and data collection.*
Outcomes	*Describe how you pre-defined primary and secondary outcome measures and how you assessed these measures.*

Dual use research of concern

Policy information about dual use research of concern

Hazards

Could the accidental, deliberate or reckless misuse of agents or technologies generated in the work, or the application of information presented in the manuscript, pose a threat to:

No	Yes	
☒	☐	Public health
☒	☐	National security
☒	☐	Crops and/or livestock
☒	☐	Ecosystems
☒	☐	Any other significant area

Experiments of concern

Does the work involve any of these experiments of concern:

No	Yes	
☒	☐	Demonstrate how to render a vaccine ineffective
☒	☐	Confer resistance to therapeutically useful antibiotics or antiviral agents
☒	☐	Enhance the virulence of a pathogen or render a nonpathogen virulent
☒	☐	Increase transmissibility of a pathogen
☒	☐	Alter the host range of a pathogen
☒	☐	Enable evasion of diagnostic/detection modalities
☒	☐	Enable the weaponization of a biological agent or toxin
☒	☐	Any other potentially harmful combination of experiments and agents

Plants

Seed stocks	*Report on the source of all seed stocks or other plant material used. If applicable, state the seed stock centre and catalogue number. If plant specimens were collected from the field, describe the collection location, date and sampling procedures.*
Novel plant genotypes	*Describe the methods by which all novel plant genotypes were produced. This includes those generated by transgenic approaches, gene editing, chemical/radiation-based mutagenesis and hybridization. For transgenic lines, describe the transformation method, the number of independent lines analyzed and the generation upon which experiments were performed. For gene-edited lines, describe the editor used, the endogenous sequence targeted for editing, the targeting guide RNA sequence (if applicable) and how the editor was applied.*
Authentication	*Describe any authentication procedures for each seed stock used or novel genotype generated. Describe any experiments used to assess the effect of a mutation and, where applicable, how potential secondary effects (e.g. second site T-DNA insertions, mosiacism, off-target gene editing) were examined.*

ChIP-seq

Data deposition

- [ ] Confirm that both raw and final processed data have been deposited in a public database such as GEO.
- [ ] Confirm that you have deposited or provided access to graph files (e.g. BED files) for the called peaks.

Data access links<br>*May remain private before publication.*	*For "Initial submission" or "Revised version" documents, provide reviewer access links. For your "Final submission" document, provide a link to the deposited data.*
Files in database submission	*Provide a list of all files available in the database submission.*
Genome browser session<br>(e.g. UCSC)	*Provide a link to an anonymized genome browser session for "Initial submission" and "Revised version" documents only, to enable peer review. Write "no longer applicable" for "Final submission" documents.*

Methodology

Replicates	*Describe the experimental replicates, specifying number, type and replicate agreement.*
Sequencing depth	*Describe the sequencing depth for each experiment, providing the total number of reads, uniquely mapped reads, length of reads and whether they were paired- or single-end.*
Antibodies	*Describe the antibodies used for the ChIP-seq experiments; as applicable, provide supplier name, catalog number, clone name, and lot number.*
Peak calling parameters	*Specify the command line program and parameters used for read mapping and peak calling, including the ChIP, control and index files used.*
Data quality	*Describe the methods used to ensure data quality in full detail, including how many peaks are at FDR 5% and above 5-fold enrichment.*
Software	*Describe the software used to collect and analyze the ChIP-seq data. For custom code that has been deposited into a community repository, provide accession details.*

Flow Cytometry

Plots

Confirm that:

- [x] The axis labels state the marker and fluorochrome used (e.g. CD4-FITC).
- [x] The axis scales are clearly visible. Include numbers along axes only for bottom left plot of group (a 'group' is an analysis of identical markers).
- [x] All plots are contour plots with outliers or pseudocolor plots.
- [x] A numerical value for number of cells or percentage (with statistics) is provided.

Methodology

Sample preparation

Cell surface staining of single cell suspensions was performed in FACS buffer (PBS with 2% FBS and 2 mM EDTA) for 20 min at room temperature. For CARs containing mouse derived scFvs,, cells were first stained with a fluorochrome-conjugated AffiniPure F(ab')2 Fragment Goat Anti-Mouse IgG, F(ab')2 fragment specific antibody (Jackson ImmunoResearch) in FACS buffer for 20 min at room temperature, then washed and blocked with 2% normal mouse serum (Sigma-Aldrich, NS03L) for 20 min at room temperature. If required, cells were afterwards stained for further cell surface markers and then resuspended in FACS buffer. CARs containing humanized scFvs were stained with fluorochrome-conjugated AffiniPure F(ab')2 Fragment Goat Anti-Human IgG, F(ab')2 fragment specific antibody (Jackson ImmunoResearch) together with further cell surface markers. Mitochondrial mass was determined using MitoTracker Orange CMTMRos (Invitrogen). Cells were first incubated with 10nM MitoTracker Orange CMTMRos in culture medium for 30 min at 37°C, then washed with PBS and incubated with antibodies for cell surface staining.

For assessment of intracellular protein levels, CAR T cells and target cells were co-cultured at an E:T ratio of 1:1 for 4 h at 37°C in the presence of 3 µg/ml brefeldin A and 2 µM monensin (both TONBOBIO). After viability and surface staining in PBS for 25 min at room temperature, cells were fixed with IC Fixation buffer (eBioscience) for 20 min at room temperature. Intracellular staining was performed in 1x Permeabilization buffer (eBioscience) for 30 min at 4°C. Cells were subsequently resuspended in FACS buffer.

*For Glucose uptake experiments, cells were either initially stimulated with Nalm6 cells for 24h at an E:T ratio of 2:1 or analyzed at the final time point of the proliferation assay as described. Cells were washed with PBS and resuspended in glucose free RPMI-1640 (Gibco) with 10% FBS supplemented with 100µg ml-1 2-NBD-Glucose (abcam, ab146200) to a cell concentration of 3*10^6 cells ml-1. Subsequently, cells were incubated for 10min at 37°C, washed with ice-cold PBS and stained extracellularly before flow cytometric analysis.*

For phosphoflow analysis, cells were seeded in cytokine free RPMI-1640 for 2h and subsequently stimulated with Nalm6 cells for 1h at an E:T ratio of 1:1. After fixation with 100µL warm 4% paraformaldehyde (PFA) for 15 min, cells were washed and permeabilized in 300µL 90% ice-cold methanol for 10 min at 4 °C and washed twice. Subsequently, cells were incubated with

unconjugated primary phospho-antibodies for 1h at room temperature, washed and stained with an AF594-coupled secondary antibody for 30 min at room temperature.
For measurement of antigen surface levels Quantum™ Simply Cellular® anti-Mouse IgG (Bangs Laboratories, INC) was performed and calculated according to manufacturer's instructions.
For CAR T cell sorts, unstimulated CAR T cells were sorted on a MA900 multi-application cell sorter (Sony) using a 100 µm sorting chip. LNGFR+ CAR T cells were sorted for indicated CAR T cell subsets defined by surface expression of CD45RA and CD62L (T_N, CD45RA+CD62L+; T_CM, CD45RA-CD62L+; T_EM, CD45RA-CD62L-; T_EFF, CD45RA+CD62L-). Sorted CAR T cell subsets were cultured overnight in fresh, cytokine-supplemented medium and further used for downstream analyses. For cell sorting, the following antibodies were used: CD3-Spark Violet538, CD45RA-BV650, CD62L-PE/Dazzle594, LNGFR-PE.
For samples obtained from in vivo experiments, Fc receptors were blocked using mouse FcR Blocking Reagent (Miltenyi Biotec, 130-092-575) in addition to the CAR and surface staining as described above. Blood samples were collected in EDTA coated microtainer and washed with cold FACS buffer. All staining steps were carried out without washing steps and finally, FACS lysis solution (BD, 349202) was added to lyse red blood cells. CountBright Absolute Counting Beads (Invitrogen) were added to the samples shortly before acquisition for cell counting.

Instrument: Cytek Aurora spectral cytometer.

Software: flowJo v10.8.0

Cell population abundance: FlowJo v10.8.0

Gating strategy: First gating was always SSC-H vs FSC-H, followed by single cell gating via SSC-A vs SSC-H. All other populations were gated based on flourescent minus one (FMO) controls or unstained samples.

☒ Tick this box to confirm that a figure exemplifying the gating strategy is provided in the Supplementary Information.

Magnetic resonance imaging

Experimental design

Design type: Indicate task or resting state; event-related or block design.

Design specifications: Specify the number of blocks, trials or experimental units per session and/or subject, and specify the length of each trial or block (if trials are blocked) and interval between trials.

Behavioral performance measures: State number and/or type of variables recorded (e.g. correct button press, response time) and what statistics were used to establish that the subjects were performing the task as expected (e.g. mean, range, and/or standard deviation across subjects).

Acquisition

Imaging type(s): Specify: functional, structural, diffusion, perfusion.

Field strength: Specify in Tesla

Sequence & imaging parameters: Specify the pulse sequence type (gradient echo, spin echo, etc.), imaging type (EPI, spiral, etc.), field of view, matrix size, slice thickness, orientation and TE/TR/flip angle.

Area of acquisition: State whether a whole brain scan was used OR define the area of acquisition, describing how the region was determined.

Diffusion MRI ☐ Used ☐ Not used

Preprocessing

Preprocessing software: Provide detail on software version and revision number and on specific parameters (model/functions, brain extraction, segmentation, smoothing kernel size, etc.).

Normalization: If data were normalized/standardized, describe the approach(es): specify linear or non-linear and define image types used for transformation OR indicate that data were not normalized and explain rationale for lack of normalization.

Normalization template: Describe the template used for normalization/transformation, specifying subject space or group standardized space (e.g. original Talairach, MNI305, ICBM152) OR indicate that the data were not normalized.

Noise and artifact removal: Describe your procedure(s) for artifact and structured noise removal, specifying motion parameters, tissue signals and physiological signals (heart rate, respiration).

Volume censoring: Define your software and/or method and criteria for volume censoring, and state the extent of such censoring.

Statistical modeling & inference

Model type and settings — *Specify type (mass univariate, multivariate, RSA, predictive, etc.) and describe essential details of the model at the first and second levels (e.g. fixed, random or mixed effects; drift or auto-correlation).*

Effect(s) tested — *Define precise effect in terms of the task or stimulus conditions instead of psychological concepts and indicate whether ANOVA or factorial designs were used.*

Specify type of analysis: ☐ Whole brain ☐ ROI-based ☐ Both

Statistic type for inference — *Specify voxel-wise or cluster-wise and report all relevant parameters for cluster-wise methods.*

(See Eklund et al. 2016)

Correction — *Describe the type of correction and how it is obtained for multiple comparisons (e.g. FWE, FDR, permutation or Monte Carlo).*

Models & analysis

n/a	Involved in the study
☐	☐ Functional and/or effective connectivity
☐	☐ Graph analysis
☐	☐ Multivariate modeling or predictive analysis

Functional and/or effective connectivity — *Report the measures of dependence used and the model details (e.g. Pearson correlation, partial correlation, mutual information).*

Graph analysis — *Report the dependent variable and connectivity measure, specifying weighted graph or binarized graph, subject- or group-level, and the global and/or node summaries used (e.g. clustering coefficient, efficiency, etc.).*

Multivariate modeling and predictive analysis — *Specify independent variables, features extraction and dimension reduction, model, training and evaluation metrics.*
