# Supplementary figures and images for: CAR-adapted PIK3CD base editing enhances T cell anti-tumor potency
Source: Nat Cancer. 2026 Jan 6;7(2):368–83. doi: 10.1038/s43018-025-01099-7 (PMC12948676; doi:10.1038/s43018-025-01099-7)

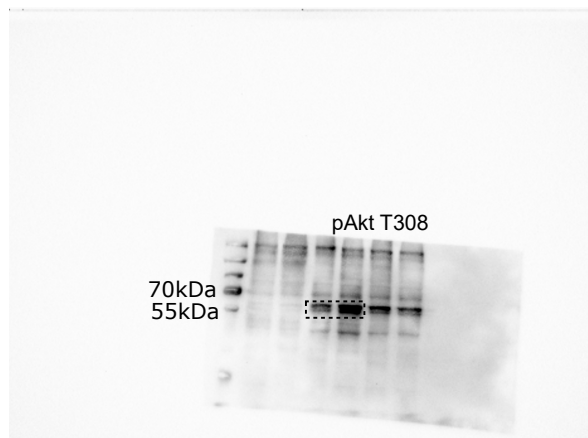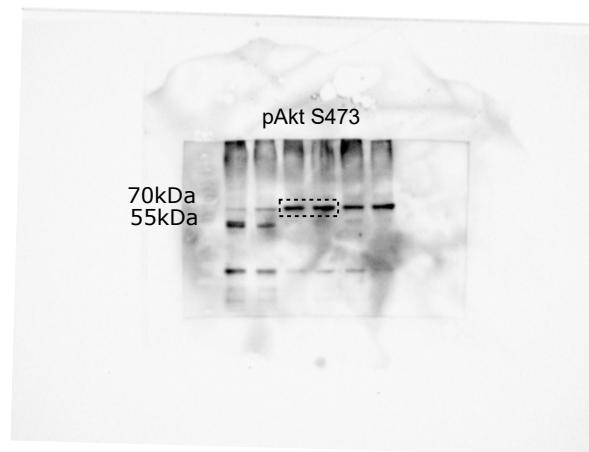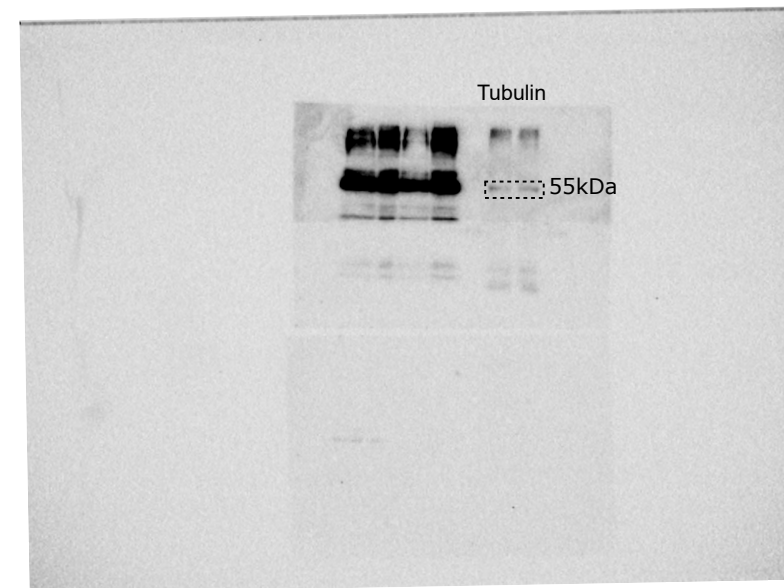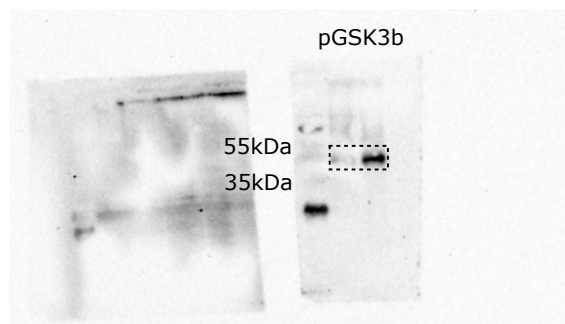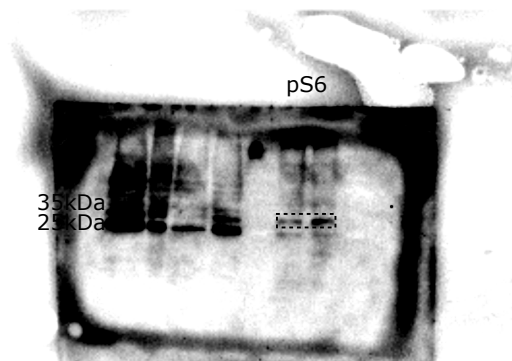

Ext. Data Fig. 2a

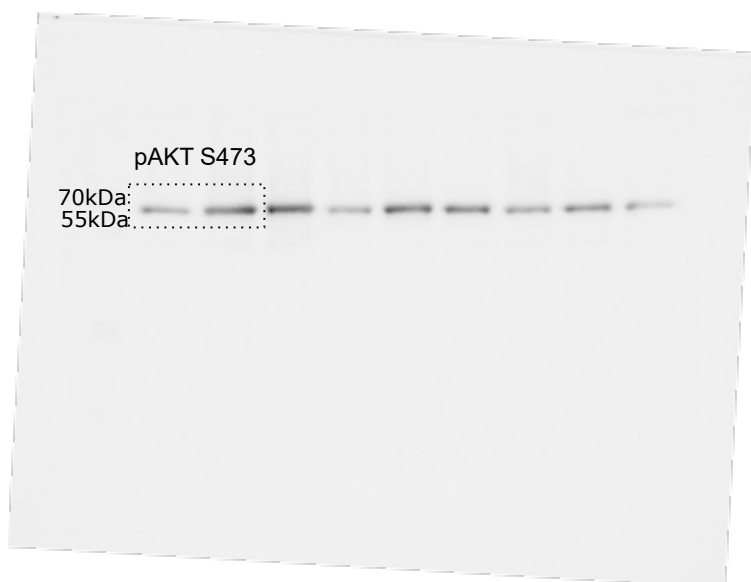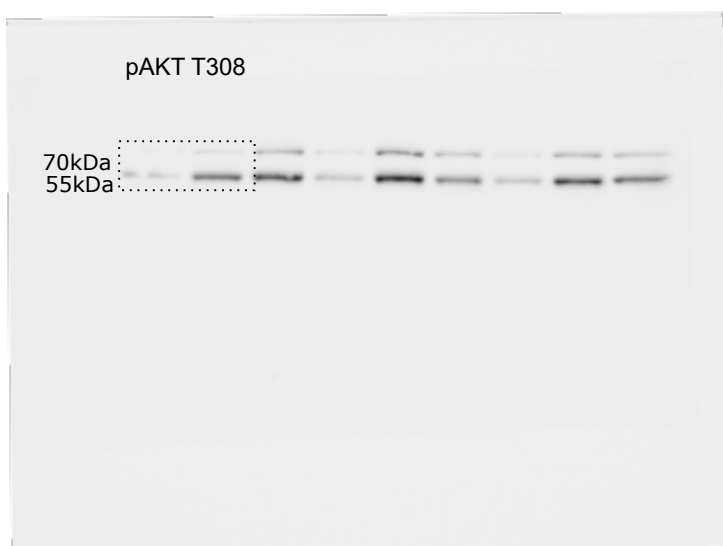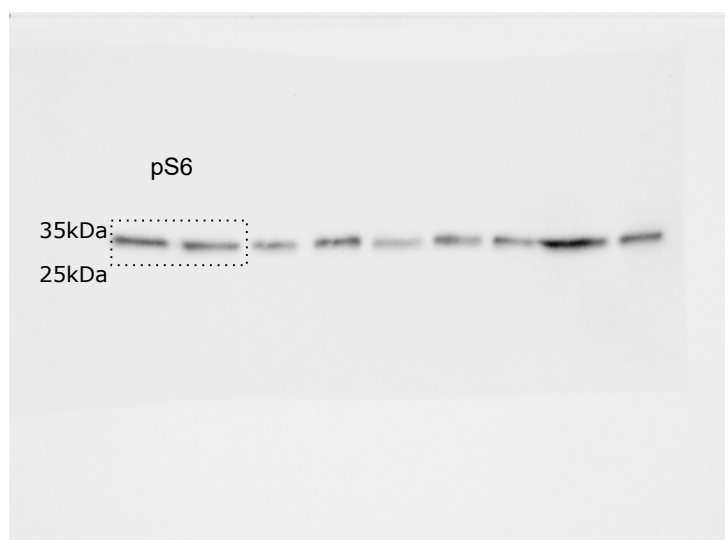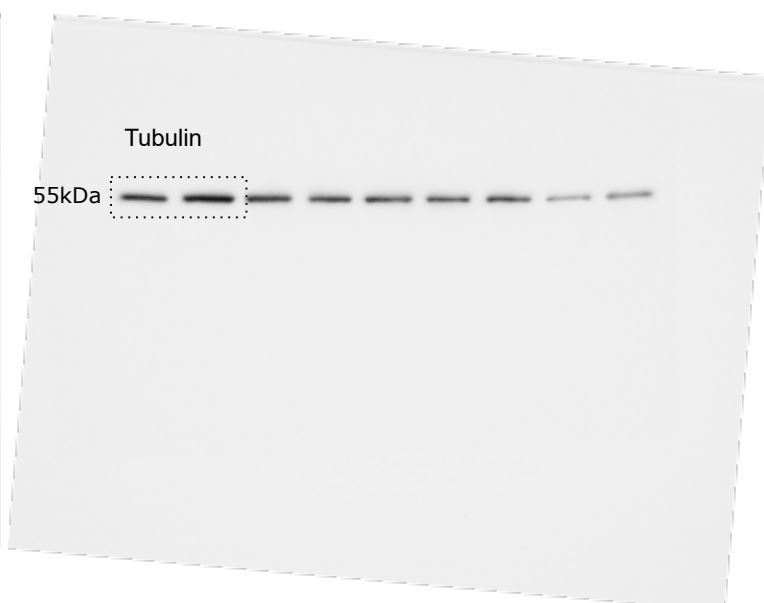

Ext. Data Fig. 2c

Supplement: Supplementary file 4 — Unprocessed western blots. [file 43018_2025_1099_MOESM4_ESM.pdf]
